# Engineering 2‐Deoxy‐D‐ribose‐5‐phosphate Aldolase for *anti*‐ and *syn*‐Selective Epoxidations of *α*,*β*‐Unsaturated Aldehydes

**DOI:** 10.1002/anie.202503054

**Published:** 2025-03-07

**Authors:** Hangyu Zhou, Andreas Kunzendorf, Guangcai Xu, Hylke O. T. Frietema, Andy‐Mark W. H. Thunnissen, Gerrit J. Poelarends

**Affiliations:** ^1^ Department of Chemical and Pharmaceutical Biology Groningen Research Institute of Pharmacy University of Groningen Antonius Deusinglaan 1 9713 AV Groningen, The Netherlands.; ^2^ Molecular Enzymology Group Groningen Institute of Biomolecular Sciences and Biotechnology University of Groningen Nijenborgh 4 9747 AG Groningen, The Netherlands

**Keywords:** *anti*-selectivity, biocatalysis, catalytic promiscuity, enzyme engineering, peroxidation

## Abstract

The enzyme 2‐deoxy‐D‐ribose‐5‐phosphate aldolase (DERA) naturally catalyzes the reversible aldol addition between acetaldehyde and D‐glyceraldehyde‐3‐phosphate to yield 2‐deoxy‐D‐ribose‐5‐phosphate. Herein we describe the redesign of DERA into a proficient non‐natural peroxygenase that promotes the asymmetric epoxidation of various *α*,*β*‐unsaturated aldehydes. This repurposed aldolase, named DERA‐EP, is able to utilize H_2_O_2_ to accomplish both *anti*‐ and *syn*‐selective epoxidations of various *α*,*β*‐unsaturated aldehydes to give the corresponding epoxides with moderate to high diastereoselectivity (diastereomeric ratio up to 99 : 1) and excellent enantioselectivity (enantiomeric ratio up to 99 : 1). Crystallographic analysis of DERA‐EP in a substrate‐free and substrate‐bound state provides a structural context for the evolved activity, a clear explanation for the high enantioselectivity, and compelling evidence for catalysis via enzyme‐bound iminium ion intermediates. The unprecedented *anti*‐selectivity of DERA‐EP with multiple *α*,*β*‐unsaturated aldehydes is complementary to the *syn*‐selectivity of previously reported enzyme‐, metal‐ and organo‐catalysts, making DERA‐EP an attractive new asset to the toolbox of epoxidation catalysts.

## Graphical abstract

**The** ***anti*** **‐ and** ***syn*** **‐selective epoxidation** of various *α*,*β*‐unsaturated aldehydes is promoted by an engineered variant of 2‐deoxy‐D‐ribose‐5‐phosphate aldolase (DERA), giving rise to various *α*,*β*‐epoxy‐aldehydes with excellent enantiopurity (enantiomeric ratio up to 99 : 1).

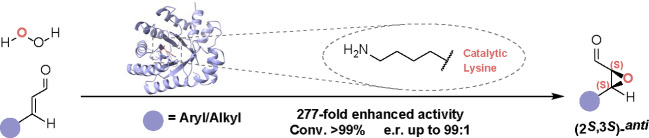

Chiral epoxides are versatile building blocks for the synthesis of biologically active compounds, fine chemicals, and pharmaceuticals.[^1^, ^2^, ^3^] The development of efficient catalysts for the synthesis of chiral epoxides has therefore received considerable attention.[^4^, ^5^, ^6^, ^7^, ^8^, ^9^, ^10^, ^11^] Interestingly, in the past two decades, remarkable progress has been made in the development of asymmetric organocatalytic epoxidation procedures.[^12^, ^13^, ^14^, ^15^, ^16^, ^17^, ^18^, ^19^, ^20^] In 2005, Jørgensen and co‐workers reported the first asymmetric organocatalytic epoxidation of *α*,*β*‐unsaturated aldehydes using peroxides as the oxidant and a chiral amine as catalyst.[^21^, ^22^] In 2011, Akagawa and Kudo developed a resin‐supported peptide catalyst which could perform this useful epoxidation reaction in aqueous media with good enantio‐ and diastereoselectivity.^[23]^ Following these pioneering discoveries in the organocatalysis field, we demonstrated that the enzyme 4‐oxalocrotonate tautomerase (4‐OT), which uses an N‐terminal proline as key catalytic residue,[^24^, ^25^] can be repurposed as a non‐natural peroxygenase promoting enantiocomplementary epoxidations of various *α*,*β*‐unsaturated aldehydes using either t‐BuOOH or H_2_O_2_ as the nucleophile.^[26]^ Furthermore, the epoxidation activity of this unique cofactor‐independent peroxygenase was further enhanced by the directed evolution of a tandem‐fused enzyme variant, enabling gram‐scale epoxide synthesis.[^27^, ^28^]

Notably, the *syn*‐product is commonly the major diastereomer produced through asymmetric epoxidations of activated alkenes promoted by either metal‐,^[29]^ organo‐,[^30^, ^31^, ^32^, ^33^, ^34^, ^35^, ^36^, ^37^] or enzyme‐catalysts,[^26^, ^27^, ^28^] and the development of new catalysts for asymmetric *anti*‐selective^[38]^ epoxidations of *α*,*β*‐unsaturated aldehydes remains an elusive goal (Figure 1). In this regard, enzyme catalysts, due to their exquisite enantio‐, regio‐, and stereoselectivity, may have a distinctive potential to advance towards this goal.^[39]^

**Figure 1:**
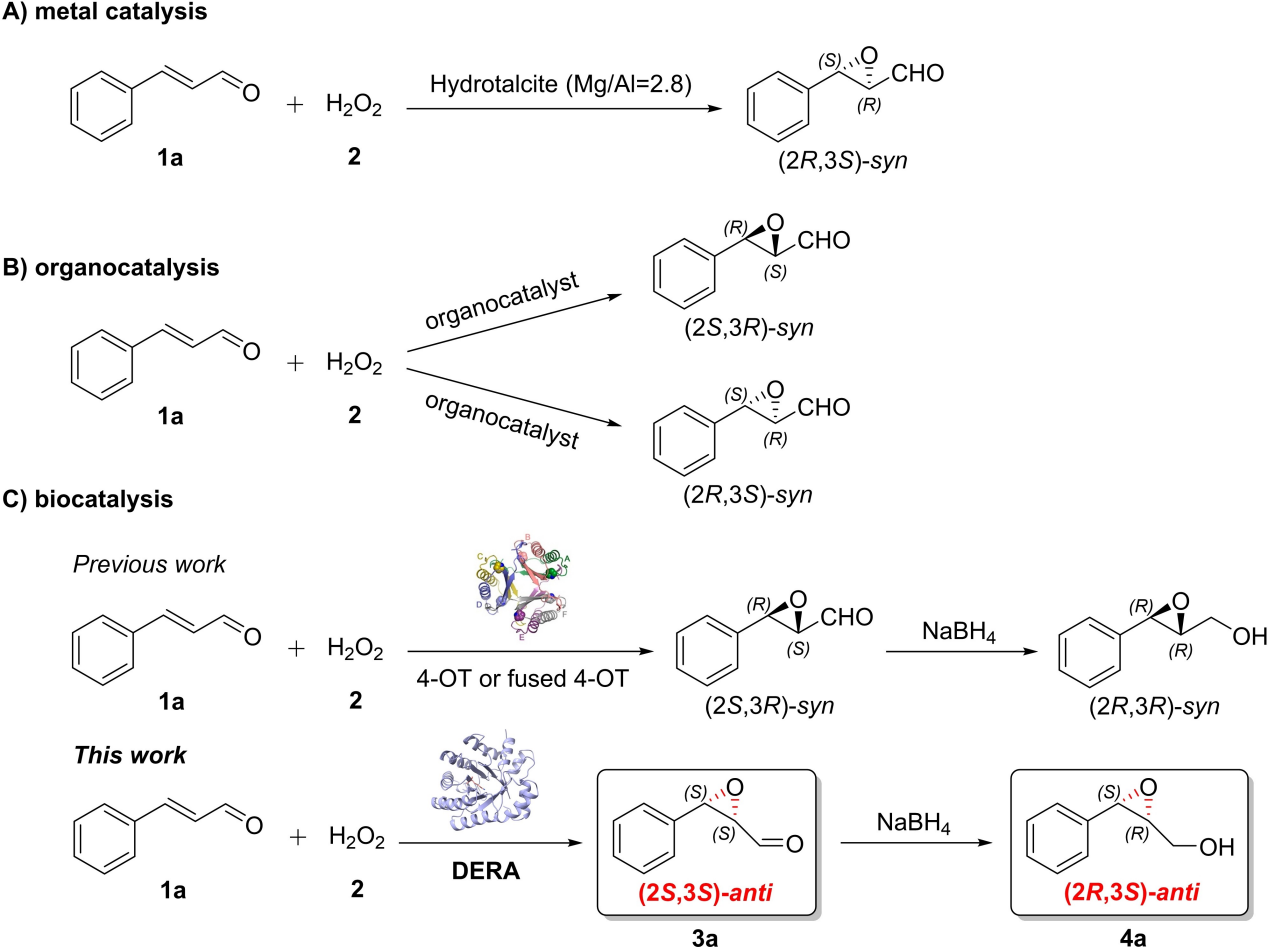
Different *α*,*β*‐epoxy‐aldehyde product configurations generated from *trans*‐cinnamaldehyde and H_2_O_2_ by (A) metal catalysis,^[29]^ (B) organocatalysis,[^30^, ^31^, ^32^, ^33^, ^34^, ^35^, ^36^, ^37^] and (C) biocatalysis.[^26^, ^27^, ^28^] Note that while the enzymatic product **3 a** has a 2*S*,3*S* configuration, the corresponding product **4 a** has a 2*R*,3*S* configuration. There is no change at the stereocenter of the epoxy alcohol, but the descriptor has changed due to different prioritization of the substituents at the C2 chiral center relative to the epoxy‐aldehyde precursor.

Recently, our laboratory demonstrated that the archetypical class I aldolase 2‐deoxy‐D‐ribose‐5‐phosphate aldolase (DERA)[^40^, ^41^] from *Escherichia coli* K‐12, can be redesigned into a proficient “Michaelase” that catalyzes the enantioselective Michael addition of nitromethane to *α*,*β*‐unsaturated aldehydes via an iminium‐based mechanism.^[42]^ These results show that DERA can accommodate both enamine catalysis[^40^, ^41^, ^43^, ^44^, ^45^, ^46^] (natural aldolase activity) and iminium catalysis.^[47]^ Combined with the ability of DERA to accept various non‐native *α*,*β*‐unsaturated aldehydes, this enzyme represents a promising scaffold to explore promiscuous stereoselective epoxidation activities.

Here, we report our protein engineering effort to repurpose DERA into a proficient cofactor‐independent unnatural peroxygenase. Remarkably, this engineered peroxygenase, named DERA‐EP, is able to utilize H_2_O_2_ to accomplish both *anti*‐ and *syn*‐selective epoxidations of a range of *α*,*β*‐unsaturated aldehydes to give the corresponding oxiranes with moderate to high diastereoselectivity (diastereomeric ratio up to 99 : 1) and excellent enantioselectivity (enantiomeric ratio up to 99 : 1). Crystallographic analysis of DERA‐EP in a substrate‐free and substrate‐bound state provides a structural context for the evolved peroxygenase activity.

We started our investigation by testing whether wild‐type DERA can promiscuously catalyze the epoxidation of cinnamaldehyde (**1 a**) with H_2_O_2_ (**2**) to give the corresponding *α*,*β*‐epoxy‐aldehyde **3 a** (Figure 1). Pleasingly, when DERA (10 μM) was incubated with 1 mM **1 a** and 20 mM **2** at room temperature, 23 % of substrate **1 a** was consumed within 20 h, and the corresponding *α*,*β*‐epoxy‐aldehyde **3 a** was identified by GC‐MS analysis. Chiral reverse‐phase HPLC analysis of the alcohol derivative of **3 a** revealed the enzymatic product to be (2*S*,3*S*)‐**3 a** with a diastereomeric ratio (d.r.) of 98 : 2 (*anti*/*syn*) and an enantiomeric ratio (e.r.) of 99 : 1. Remarkably, this *anti*‐selective epoxidation activity of DERA stands in contrast with the *syn*‐selective epoxidation reactions catalyzed by previously reported metal‐,^[29]^ organo‐[^30^, ^31^, ^32^, ^33^, ^34^, ^35^, ^36^, ^37^] and enzyme‐catalysts,[^26^, ^27^, ^28^] providing a new route to access *anti*‐epoxides (Figure 1).

In order to enhance the promiscuous peroxygenase activity of DERA, we started a directed evolution[^48^, ^49^, ^50^] campaign. For this, a previously constructed library of DERA mutants^[42]^ was screened to identify mutations that give improved peroxygenase activity (Table S3). To minimize the screening effort, we used a solid‐phase pre‐screening assay termed as activated iminium colony staining (AICS) to identify colonies producing active enzyme variants.^[51]^ This AICS assay makes use of the iminium‐activated colorimetric “turn‐on” probe 2‐hydroxycinnamaldehyde, which upon complexation with the catalytic lysine residue of DERA gives a brightly colored merocyanine‐dye‐type structure. DERA variants that formed this brightly colored species upon incubation with the probe proved to have a substantial epoxidation activity, whereas variants that showed no staining by the probe exhibited no or very low‐level epoxidation activity.^[51]^ Approximately 890 bacterial colonies were selected and the enzyme variants were screened for enhanced epoxidation activity by following the depletion of **1 a** using a spectrophotometric assay. The best variant, 4B11, showed an ∼26‐fold enhanced activity over wild‐type DERA (Figure 2). To further improve the peroxygenase activity of DERA (4B11), we applied site‐saturation mutagenesis (SSM)^[52]^ achieved by QuickChange PCR (Table S3).[^53^, ^54^, ^55^] The amino acid positions mutated were chosen mainly based on their close proximity (maximum 5 Å distance) to the active site, according to the crystal structure of cinnamaldehyde‐bound DERA‐MA, the best DERA variant for the nitromethane addition reaction (PDB entry 7P76).^[42]^ Additionally, it has been reported that position 21 is located at the entrance to the active site and that mutations at position 17 improve the aldolase activity.^[56]^ Together with the existing mutated positions in variant 4B11, a total of 36 amino acid positions were targeted by SSM in the second round of our directed evolution campaign. The best mutant in this round, 1E1, increased the peroxygenase activity by ~3‐fold compared to 4B11 (Figure 2). Mutant 2A7 was obtained after two extra rounds of SSM (Table S3). In the last round, we targeted positions 48 and 49 of the 2A7 variant, yielding the DERA‐EP mutant (11A8) with a total of 14 amino acid substitutions compared to the parental DERA enzyme (Table S4). The DERA‐EP variant showed a 277‐fold enhancement in catalytic activity for the epoxidation of **1 a** compared to the wild‐type enzyme (Figure 2). While this tailor‐made DERA variant retained the unique *anti*‐selectivity, a gradual decrease in the diastereoselectivity of the evolved enzyme variants during the directed evolution trajectory was observed, resulting in a decrease in the d.r. value of the product from 98 : 2 to 63 : 37 (Figure 2B). Pleasingly, all engineered enzyme variants retained excellent enantioselectivity, giving highly enantioenriched product with an e.r. value of 99 : 1 (Figure 2B). To further identify the absolute configuration of the product, we performed chiral normal‐phase HPLC according to a literature procedure,^[57]^ which showed the respective retention times of the four *α*,*β*‐epoxy‐aldehyde stereoisomers (Figure S14). This analysis revealed the enzymatic enantiocontrol over both carbons of the double bond of **1 a**, resulting in the formation of the *anti*‐(2*S*,3*S*)*‐* **3 a** (e.r. of 99 : 1, d.r. of 63 : 37, Table 1).

**Figure 2:**
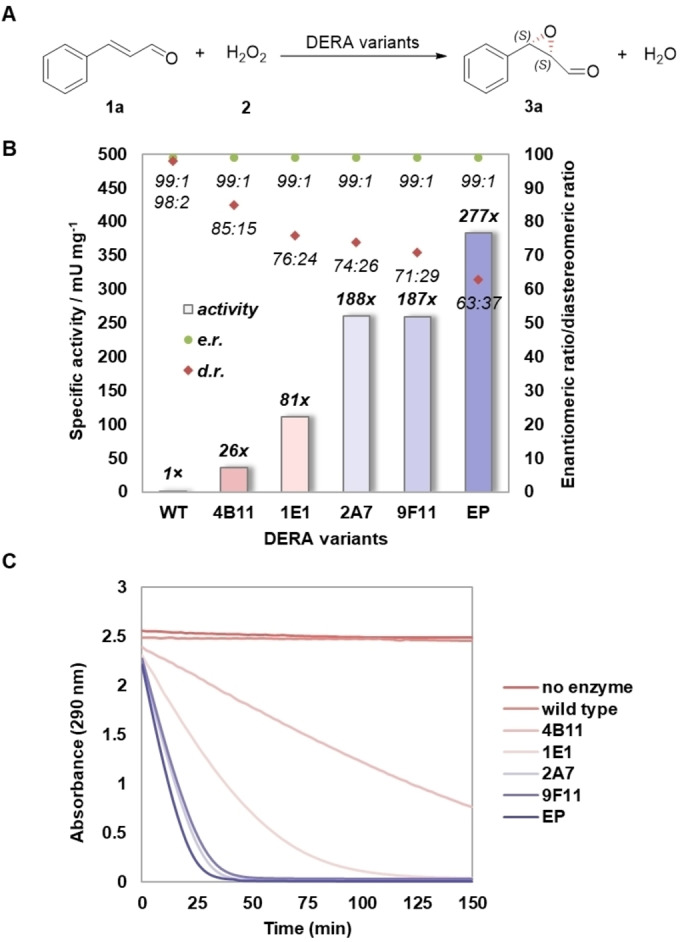
Epoxidation activity of DERA variants. A) Reaction scheme of the DERA‐catalyzed epoxidation reaction of **1 a** with **2** to yield *α*,*β*‐epoxy‐aldehyde **3 a**. B) Comparison of the peroxygenase activity (in mU/mg of protein) of wild‐type DERA (WT) and engineered DERA variants. C) Reaction progress curves of different DERA variants. Reaction conditions: 1 mM **1 a**, 20 mM **2**, 5 μM DERA variant, 20 mM HEPES, 100 mM NaCl, pH 6.5 with 5 % (v/v) EtOH. The specific activity of DERA‐EP is 383 mU per mg of protein.

**Table 1:** Substrate scope of DERA‐EP.^[a]^

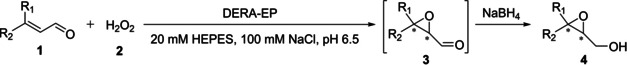								
Entry	1	R_1_	R_2_	t[h]^[c]^	Conv.^[d]^	e.r.^[e]^	d.r.^[f]^	Abs. config. of product **4**
1	**a**	H	Ph	0.5	99	99 : 1	63 : 37	2*R*, 3*S* (*anti*)*
2^[b]^	**a**	H	Ph	28	99	99 : 1	84 : 16	2*R*, 3*S* (*anti*)*
3	**b**	H	*o*‐Cl‐Ph	0.5	99	99 : 1	85 : 15	2*S*, 3*S* (*syn*)^[g]^
4	**c**	H	*m*‐Cl‐Ph	0.6	99	99 : 1	72 : 28	2*S*, 3*S* (*syn*)^[g]^
5	**d**	H	*p*‐Cl‐Ph	1	99	n.d.	94 : 6	2*R*, 3*S* (*anti*)^[h]^
6	**e**	H	*p*‐F‐Ph	0.6	99	n.d.	98 : 2	2*R*, 3*S* (*anti*)^[h]^
7	**f**	H	*p*‐Br‐Ph	0.6	99	99 : 1	99 : 1	2*R*, 3*S* (*anti*) ^[h]^
8	**g**	H	*o*‐NO_2_‐Ph	1	99	99 : 1	76 : 24	2*S*, 3*S* (*syn*) ^[g]^
9	**h**	H	*p*‐NO_2_‐Ph	0.6	99	n.d.	81 : 19	2*R*, 3*S* (*anti*) ^[h]^
10	**i**	H	*o*‐CH_3_‐Ph	2	99	99 : 1	99 : 1	2*R*, 3*S* (*anti*) ^[h]^
11^[i]^	**j**	Me	(CH_3_)_2_CCHCH_2_CH_2_	6	78	99 : 1	82 : 18	2*R*, 3*S* (*anti*) ^[h]^
12	**k**	H	*m*‐OCH_3_‐Ph	2	99	99 : 1	60 : 40	2*R*, 3*S* (*anti*) ^[h]^

[a] Analytical scale reactions were performed with 1 mM **1 a**–**k**, 20 mM **2**, and 5 μM DERA‐EP in 20 mM HEPES, 100 mM NaCl, pH 6.5, 5 % v/v EtOH (ACN for **1 f**, **1 g** and **1 h**) at room temperature, reaction volume=4 mL, enzymatic product **3** was reduced with NaBH_4_ to **4** after completion of the reaction. [b] 100 mM *t*‐BuOOH was applied instead of 20 mM **2**. [c] Reaction times determined after UV/Vis analysis indicated consumption of **1 a**–**k**. [d] Conversion into the epoxide product determined by GC‐MS. [e] The e.r. value of the major diastereomer, determined by chiral reverse‐phase HPLC. [f] Major diastereomer/minor diastereomer, determined by chiral reverse‐phase HPLC. [g] Absolute configuration determined by chiral reverse‐phase HPLC using chemically synthesized (2*S*,3*S*)‐ and (2*R*,3*R*)‐*syn* isomers. [h] Relative configuration determined by chiral reverse‐phase HPLC using a chemically synthesized mixture of rac‐*syn* and rac‐*anti* isomers as well as chemically synthesized *syn* isomers. The absolute configuration is tentatively assigned based on analogy. [i] **1 j**: *E*/*Z*=3 : 2. n.d.: not determined. *Absolute configuration determined by chiral normal‐phase HPLC using a chemically synthesized mixture of rac‐*syn* and rac‐*anti* isomers and previously reported chiral HPLC data.^[57]^

Furthermore, we have tested the use of *t*‐BuOOH as the oxidant instead of H_2_O_2_. The outcome shows that the use of *t*‐BuOOH results also in the enrichment of the *anti‐*2*S*,3*S*‐product **3a** (d.r. of 84 : 16, e.r. of 99 : 1, Table 1, Figure S15); hence, there is no change in the stereochemical outcome based on the use of a different oxidant. Notably, *t*‐BuOOH is a rather poor substrate for the enzyme, necessitating prolonged incubation to achieve full conversion (Table 1).

With an improved variant in hand, we next investigated the carbonyl substrate scope of DERA‐EP in the asymmetric epoxidation reaction. As shown in Table 1, high conversions (up to 99 %) were achieved for a broad range of aromatic *α*,*β*‐unsaturated aldehydes, bearing either electron‐withdrawing or electron‐donating substitutions in the *ortho*‐, *meta*‐, or *para*‐position of the aromatic ring (**1 b**–**1 i, 1 k**), furnishing the corresponding *α*,*β*‐epoxy aldehydes (**3 b**–**3 i, 3 k**) in moderate to excellent diastereo‐ and enantiopurity (d.r. of 60 : 40–99 : 1, e.r. of 99 : 1; Figures S17–S27). In addition to aromatic cinnamaldehyde derivatives, DERA‐EP also accepts the aliphatic aldehyde citral (**1 j**), achieving good conversion (78 %) and excellent product enantiopurity (e.r. of 99 : 1, Table 1 and Figure S26). Interestingly, while unique *anti* diastereomers were obtained as the main product when *ortho*‐methyl‐, *meta*‐methoxy‐, and *para*‐substitutions were present on the cinnamaldehyde substrates, *ortho*‐chloro‐, *meta*‐chloro‐ and *ortho*‐nitro‐substitutions gave rise to *syn* diastereomers as the main product (Table 1). The results further show that DERA‐EP has a strong preference for cinnamaldehyde derivatives, while showing very low‐level activity towards benzylideneacetone and its *para*‐chloro derivative and no activity towards other structurally distinct enals and enones (Figure S1).

The biocatalytic potential of DERA‐EP was further demonstrated by performing semi‐preparative scale synthesis with the substrates **1 a**–**1 h** and **1 j** (Table 2). NMR and chiral reverse‐phase HPLC results (for details, see Supporting Information) showed that DERA‐EP was able to perform the synthesis (at 100 milligram‐scale) of the epoxy‐aldehydes **3 a–3 h** and **3 j** (reaction time of 15–240 min) in moderate to good yields (74–89 %, 83–125 mg), with high conversions (up to 96 %) and excellent enantiopurity (e.r. up to 99 : 1). Pleasingly, with *p*‐chloro‐cinnamaldehyde (**1 d**), *p*‐fluoro‐cinnamaldehyde (**1 e**), *p*‐bromo‐cinnamaldehyde (**1 f**), *p*‐nitro‐cinnamaldehyde (**1 h**) and citral (**1 j**) as substrate, the *anti*‐products were obtained with moderate to good diastereopurity (d.r. of 76 : 24–91 : 9). While a small decrease in product diastereomeric ratio was observed for most substrates under these semi‐preparative scale conditions, the enzymatic reaction with substrate **1 a** resulted in an almost complete loss of diastereoselectivity (Table 2, entry 1). This prompted us to test several other DERA variants from earlier rounds of the directed evolution campaign (Table S3) for their ability to produce diastereomerically enriched epoxide product **3 a**. Gratifyingly, using the same semi‐preparative scale conditions, DERA 2A1 was found to give the *anti*‐product **3 a** (reaction time=24 h) with excellent enantiopurity (e.r. of 99 : 1) and acceptable diastereopurity (d.r. of 72 : 28, Figure S5). These results underscore the potential to engineer synthetically useful *anti*‐selective DERA variants for epoxide production.

**Table 2:** Semi‐preparative scale DERA‐EP‐catalyzed epoxidations of *α*,*β*‐unsaturated aldehydes using H_2_O_2_ as oxidant.^[a]^

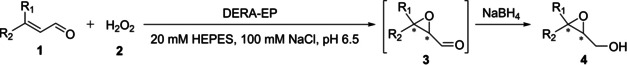										
Entry	1	R^1^	R^2^	t[min]^[b]^	Conv.^[c]^	TOF[min^−1^]	Yield[%]^[d]^	e.r.^[e]^	d.r.^[f]^	Abs. config. of product **4**
1	**a**	H	Ph	20	99	9.9	75 (83 mg)	99 : 1	52 : 48	2*R*, 3*S* (*anti*)*
2	**b**	H	*o*‐Cl‐Ph	15	99	13.2	85 (115 mg)	99 : 1	82 : 18	2*S*, 3*S* (*syn*)^[g]^
3	**c**	H	*m*‐Cl‐Ph	40	99	5.0	89 (120 mg)	99 : 1	65 : 35	2*S*, 3*S* (*syn*)^[g]^
4	**d**	H	*p*‐Cl‐Ph	30	96	6.4	77 (101 mg)	n.d.	88 : 12	2*R*, 3*S* (*anti*)^[h]^
5	**e**	H	*p*‐F‐Ph	90	99	2.2	86 (106 mg)	n.d.	76 : 24	2*R*, 3*S* (*anti*)^[h]^
6	**f**	H	*p*‐Br‐Ph	70	97	2.8	74 (125 mg)	95 : 5	91 : 9	2*R*, 3*S* (*anti*)^[h]^
7	**g**	H	*o*‐NO_2_‐Ph	35	99	5.7	86 (124 mg)	99 : 1	70 : 30	2*S*, 3*S* (*syn*)^[g]^
8	**h**	H	*p*‐NO_2_‐Ph	80	99	2.5	81 (116 mg)	n.d.	80 : 20	2*R*, 3*S* (*anti*)^[h]^
9	**j** ^[i]^	Me	(CH_3_)_2_CCHCH_2_CH_2_	240	76	0.6	83 (80 mg)	95 : 5	82 : 18	2*R*, 3*S* (*anti*)^[h]^

[a] Assay conditions: reaction mixture consisted of 5 mM **1**, 40 mM **2**, and 25 μM DERA‐EP in 20 mM HEPES, 100 mM NaCl, pH 6.5, 5 % v/v EtOH (ACN for **1 f, 1 g** and **1 h**) at room temperature, volume=150 mL. [b] Reaction times determined after UV/Vis analysis indicated consumption of **1 a**–**h** and **1 j**. [c] Conversion determined by NMR. [d] Yield of the diastereomer mixture. [e] Major diastereomer, determined by chiral reverse‐phase HPLC. [f] Major diastereomer/minor diastereomer, determined by NMR. [g] Absolute configuration determined by chiral reverse‐phase HPLC using chemically synthesized (2*S*,3*S*)‐ and (2*R*,3*R*)‐*syn* isomers. [h] Relative configuration determined by chiral reverse‐phase HPLC using a chemically synthesized mixture of rac‐*syn* and rac‐*anti* isomers as well as chemically synthesized *syn* isomers. The absolute configuration is tentatively assigned based on analogy. * Absolute configuration determined by chiral normal‐phase HPLC using a chemically synthesized mixture of rac‐*syn* and rac‐*anti* isomers and previously reported chiral HPLC data.^[57]^. [i] **1 j**: *E*/*Z*=3 : 2. n.d.: not determined.

To provide a structural context for the evolved peroxygenase activity, we determined the crystal structure of DERA‐EP at 1.40 Å resolution (Figure 3, Table S5).^[58]^ Like in the previously determined structure of DERA‐MA,^[42]^ the mutations in DERA‐EP occur in strand‐helix connecting loops surrounding the active site at the C‐terminal end of the TIM barrel, except for mutation T197S, which is located at the opposite side of the barrel (Figure 3A). Nine of the fourteen mutations are identical to those in DERA‐MA, and the overall conformations of the two DERA variants are highly similar (Figure 3B). Compared to wild‐type DERA, three loops in DERA‐EP with mutations display a different conformation, i. e. the β1‐α2 loop (containing mutations T18S, L20S, D22G and D24Y), the β7‐α8 loop (containing mutations P202V, A203T and R207S) and the β8‐α9 loop (containing mutations G236S and S239S). The β1‐α2 loop reveals the largest conformational difference, with residues 20 and 21 being displaced from the active site. No clear density is observed for these residues in the DERA‐EP structure, signifying that this part of the β1‐α2 loop, including mutation L20S, becomes highly disordered. In DERA‐MA, the β1‐α2 loop, although less disordered, exhibits a similar displacement, opening up a pocket at the active site suitable for binding cinnamaldehyde.^[42]^ To investigate substrate binding in DERA‐EP, we determined two additional high‐resolution crystal structures obtained by briefly soaking DERA‐EP crystals with 4‐chloro‐cinnamaldehyde and 4‐nitro‐cinnamaldehyde (Table S5).^[58]^ The electron density maps clearly reveal the presence of the Schiff base reaction intermediates formed between Lys‐167 and the cinnamaldehyde derivatives (Figures 3C, D). Their modes of binding are highly similar to that of cinnamaldehyde in the DERA‐MA variant (PDB entry 7P76),^[42]^ with the phenyl ring and C3 carbon of the substrates forming van der Waals contacts with the side chains of Tyr‐49, Val‐73 and Phe‐76. These residues line the pocket opened up by the displacement of the β1‐α2 loop. Tyr‐49 provides additional stabilization of the reaction intermediates via favorable interactions between its phenyl ring and the cinnamaldehyde ring substituents. Arg‐172 stabilizes Tyr‐49 via a hydrogen bond interaction, possibly explaining the activity enhancing effect of the K172R mutation. The *para*‐nitro substituent is further stabilized through a hydrogen bond with the main chain NH of Tyr‐49, while the *para*‐chlorine substituent may form a favorable halogen interaction with Asp‐23. Thus, none of the mutated residues in DERA‐EP is directly involved in substrate binding. Rather, some of the mutations induce a partial reorganization of the active site, enabling productive binding of the cinnamaldehyde derivatives close to Lys‐167 and thereby facilitating the formation of the initial Schiff base reaction intermediate.

**Figure 3:**
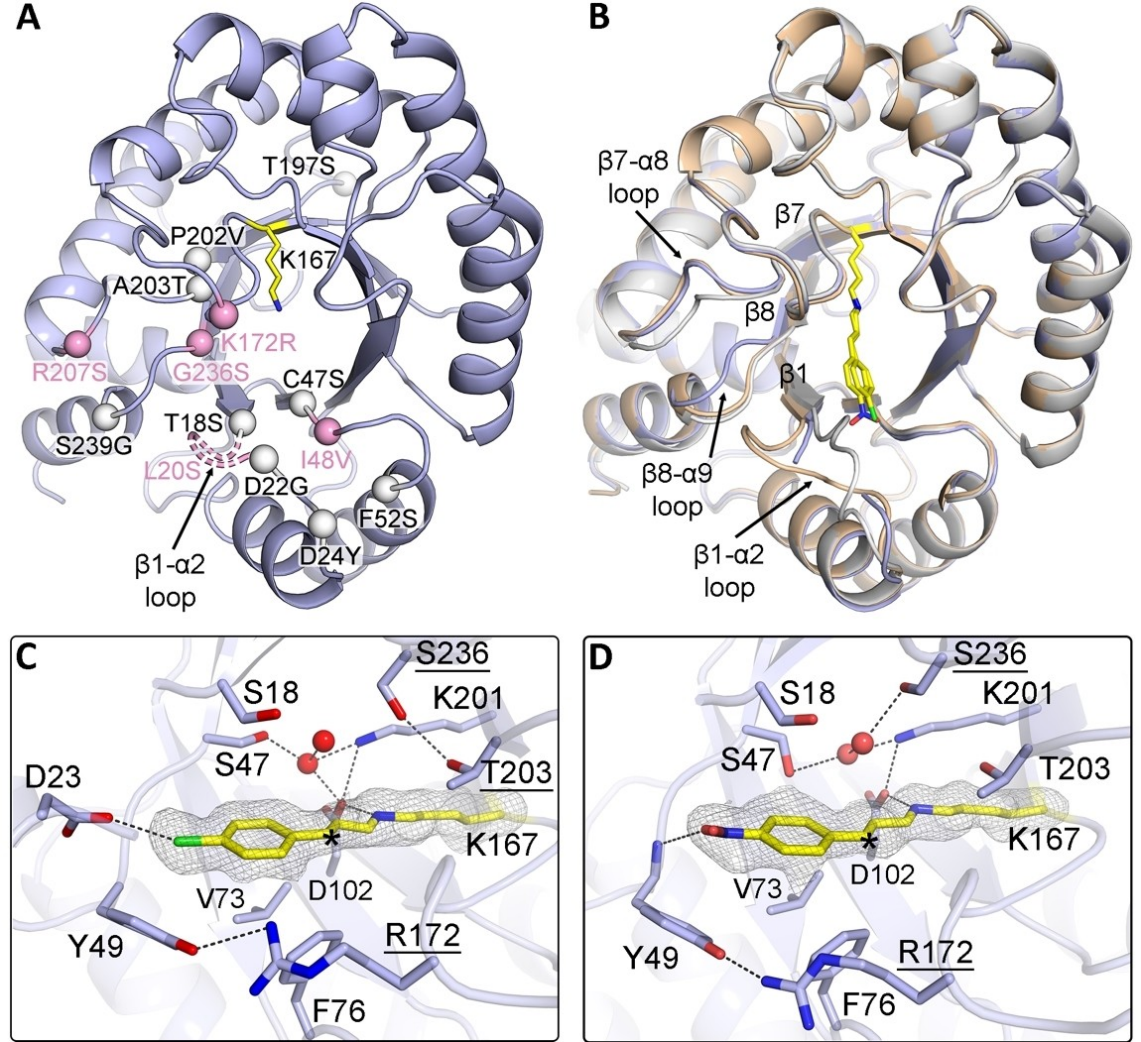
Crystal structures and active site of DERA‐EP. A) Overall structure of apo DERA‐EP (this study, PDB entry 9FD7) in cartoon representation. The fourteen mutations in the DERA‐EP polypeptide chain are indicated as spheres. Mutations also present in DERA‐MA are in white color, the others in pink. Mutation L20S is not observed in the structure due to disorder of the β1‐α2 loop. B) Overlay of the overall structures of DERA‐EP (light blue), DERA‐MA (wheat, PDB entry 7P75) and wild‐type DERA (light grey, PDB entry 1P1X). Also shown in yellow sticks are the two Schiff base reaction intermediates formed between Lys‐167 and the 4‐chloro‐ and 4‐nitro‐cinnamaldehyde derivatives. C,D) Active sites in 4‐chloro‐cinnamaldehyde‐soaked and 4‐nitro‐cinnamaldehyde‐soaked DERA‐EP (this study, PDB entries 9FD8 and 9FD9, respectively), showing the covalent Schiff base intermediates formed with Lys‐167 (yellow) in stick representation. Active site residues of DERA‐EP are in light blue. The grey mesh represents the electron densities for the Schiff base reaction intermediates (*F*_o_‐*F*_c_ Fourier omit maps, contoured at 2.5σ). Waters are represented as red spheres. Hydrogen bonds are shown as black dashed lines. The dashed line between the 4‐chloro substituent and Asp‐23 represents a halogen interaction.

The substrate‐bound DERA‐EP crystal structures also provide a clear explanation why subsequent addition of hydrogen peroxide to the C3 carbon atom of the reaction intermediate is *R*‐stereoselective. The *si*‐face of the C3 carbon is shielded due to contacts with Val‐73 and Phe‐76, while its *re*‐face is solvent accessible. The region in the active site of DERA‐EP from which hydrogen peroxide can attack the reaction intermediate is rather hydrophilic and includes several mutations: T18S, C47S, A203T and G236S. It seems plausible that this region facilitates binding of hydrogen peroxide and promotes its attack on the C3 carbon atom, resulting in the formation of the C3−O bond. Upon ring closure and subsequent hydrolysis of the epoxy iminium ion, the chiral epoxide product is formed. The *anti*‐product presumably is formed by rotation of the C2−C3 bond after formation of the C3−O bond, but before ring closure. Unfortunately, the crystal structures do not provide evidence why such a rotation is favored for the *ortho*‐methyl‐ and *para*‐substituted substrates.

In conclusion, using multiple rounds of directed evolution, we have redesigned DERA into a non‐natural cofactor‐independent peroxygenase (named DERA‐EP), which is able to promote the epoxidation of various aldehydes (**1 a**–**1 k**) using H_2_O_2_ (**2**) as the oxidant.^[59]^ Remarkably, in addition to *syn*‐selective epoxidations, DERA‐EP is capable to promote the unprecedented *anti*‐selective epoxidation of several *α*,*β*‐unsaturated aldehydes. This biocatalytic methodology for *anti*‐selective oxirane formation is complementary to previously developed *syn*‐selective enzyme‐,[^26^, ^28^] metal‐^[29]^ and organocatalysts.[^30^, ^31^, ^32^, ^33^, ^34^, ^35^, ^36^, ^37^] An extended collection of (bio)catalysts for epoxide synthesis is important because epoxides are interesting building blocks that can be expanded into various valuable compounds,[^30^, ^60^, ^61^, ^62^, ^63^, ^64^, ^65^, ^66^, ^67^, ^68^, ^69^, ^70^] such as 2‐hydroxyalkyl furanes^[71]^ and aromatic 1,2,3‐*prim,sec,sec*‐triols.[^72^, ^73^]

With the capacity to promote both enamine and iminium catalysis, DERA represents an attractive starting point to evolve new enzymes for carbonyl transformations, with the fundamental aim to expand the enzyme toolbox for the preparation of important chiral synthons. We are currently exploring DERA and its variants for a variety of bond‐forming reactions, and further synthetically useful activities of this promiscuous protein scaffold will be reported in due course.

## Conflict of Interests

The authors declare no conflict of interest.

## Supporting information

As a service to our authors and readers, this journal provides supporting information supplied by the authors. Such materials are peer reviewed and may be re‐organized for online delivery, but are not copy‐edited or typeset. Technical support issues arising from supporting information (other than missing files) should be addressed to the authors.

Supporting Information

## Data Availability

The data that support the findings of this study are available in the supplementary material of this article.
