# Supplementary material for: Engineering 2‐Deoxy‐D‐ribose‐5‐phosphate Aldolase for anti‐ and syn‐Selective Epoxidations of α,β‐Unsaturated Aldehydes
Source: Angew Chem Int Ed Engl. 2025 Mar 7;64(20):e202503054. doi: 10.1002/anie.202503054 (PMC12070355; doi:10.1002/anie.202503054)
Supplement: Supplementary file 1 — Supporting Information [file ANIE-64-e202503054-s001.pdf]

Angewandte Chemie
Eine Zeitschrift der Gesellschaft Deutscher Chemiker

Supporting Information

Engineering 2-Deoxy-D-ribose-5-phosphate Aldolase for *anti*- and *syn*-Selective Epoxidations of *α,β*-Unsaturated Aldehydes

*H. Zhou, A. Kunzendorf, G. Xu, H. O. T. Frietema, A.-M. W. H. Thunnissen, G. J. Poelarends**

Supporting Information

Engineering 2-Deoxy-D-ribose-5-phosphate Aldolase for *anti*- and *syn*-Selective Epoxidations of *α,β*-Unsaturated Aldehydes

Hangyu Zhou[1], Andreas Kunzendorf[1], Guangcai Xu[1], Hylke O.T. Frietema[2], Andy-Mark W. H. Thunnissen[2] and Gerrit J. Poelarends[1]*

[1]Department of Chemical and Pharmaceutical Biology, Groningen Research Institute of Pharmacy, University of Groningen, Antonius Deusinglaan 1, 9713 AV Groningen, The Netherlands.

[2]Molecular Enzymology Group, Groningen Institute of Biomolecular Sciences and Biotechnology, University of Groningen, Nijenborgh 4, 9747 AG Groningen, The Netherlands

*Address correspondence to Prof. Dr. Gerrit J. Poelarends. Tel: +31-50-3633354; Fax: +31-50-3633000; E-mail: g.j.poelarends@rug.nl.

Contents

1. General information 3
2. Site-saturation mutagenesis 3
3. Screening 6
4. Enzyme expression and purification 7
5. Enzyme activity assay 8
6. Substrate scope analysis of DERA-EP 9
7. Additional potential substrates tested 10
8. Semi-preparative scale (hundred milligram) synthesis 11
9. Directed evolution overview 12
10. Proposed mechanism 14
11. X-ray crystallography 14
12. DNA and Protein Sequences 17
13. NMR Spectra 18
14. Normal-phase Chiral HPLC Analysis 28
15. Reverse-phase Chiral HPLC Analysis 29
16. Supplementary References 45

1. General information

All oligonucleotide primers used in this study (summarized in Table S1) were purchased from Eurofins Genomics. Cloning enzymes were purchased from Thermo Fisher Scientific or New England Biolabs. DNA was purified using a PCR purification kit (QIAquick®, QIAGEN). Proteins were analyzed by SDS-PAGE on precast gels (NuPAGE™ 10% Bis-Tris protein gels). Substrate **1i** was prepared according to previously published procedures using the Wittig reaction and its ^{1}H-NMR spectra match with earlier reported data.[1,2] The racemic reference compounds were synthesized according to literature procedures.[3] Other aldehyde substrates and chemicals were purchased from Sigma-Aldrich Chemical Co. (St. Louis, MO, USA), BLD Pharmatech Ltd. (Shanghai, China), TCI Europe N.V. (Zwijndrecht, Belgium) or Thermo Fisher Scientific (Geel, Belgium). ESI-MS analysis of purified enzyme variants was performed by the Mass Spectrometry core facility of the University of Groningen. Spectrophotometric measurements were performed on a V-650 or V-660 spectrophotometer from Jasco (IJsselstein, The Netherlands), and measurements in a 96-well format were performed on a SPECTROstar Omega plate reader (BMG LABTECH, Isogen Life Science, de Meern, The Netherlands). Chiral reverse-phase high performance liquid chromatography (HPLC) was performed with a Shimadzu LC-20AD HPLC with a Shimadzu SPD-M20A diode array detector or SPD-20A uv/vis detector. Chiral normal-phase HPLC was performed with a Shimadzu LC-10AT HPLC with a Shimadzu SPD-M10A diode array detector. GC-MS was conducted with a Shimadzu GC-MS-QP2010 SE. NMR spectra were recorded on a Bruker 500 MHz machine at the Drug Design Laboratory of the University of Groningen. Chemical shifts (δ) are reported in parts per million (ppm).

2. Site-saturation mutagenesis

A collection of DERA variants was constructed previously[4] and used directly for the first round of activity screening. Residues from the best mutant of the first screening round and located in the active site or in the access to the active site[5] were targeted by site-saturation mutagenesis (Table S3).[6] The DNA libraries were constructed by QuickChange PCR[7] according to an earlier reported procedure.[4] For the polymerase chain reactions, pET26b(+) containing the DERA gene served as a template. Primers containing degenerate NNK codons (Table S1) were used for the PCR reactions and each site was targeted individually. The whole length plasmid was amplified using Q5 High-Fidelity 2× Master Mix (New England Biolabs,

Ipswich, MA, USA) in a 50 µL reaction volume. The following PCR program was used: 98 °C, 30 s (initial denaturation), followed by 18 cycles of 98 °C for 10 s, 60 °C (modified according to the primers Tm) for 30 s, 72 °C for 3 min, and a final elongation step at 72 °C for 5 min. Subsequently, the PCR product was purified by using the PCR purification kit, and 1 µL of DpnI (Thermo Fisher Scientific, Waltham, MA, USA) was added to the reaction mixture and incubated at 37 °C for 1 h. The obtained DNA was used to transform *E. coli* DH5α chemically-competent cells, which were spread on LB agar plates supplemented with 30 µg/mL kanamycin. After outgrowth overnight, the colonies were combined for plasmid DNA isolation to obtain the DNA library for screening.

Table S1. Primers used for libraries construction.

Round	Name	Primer Sequence (5' →3')
2，3，4	HZH-4B11-16NNKr	TCAGGGTGGACAG**MNN**CATCAATTTCAGTGCACGCA
2，3，4	HZH-4B11-16NNKf	CACTGAAATTGATG**NNK**CTGTCCACCCTGAATGGCG
2，3，4	HZH-4B11-17NNKr	GCCATTCAGGGTGGA**MNN**GTCCATCAATTTCAGTGCACG
2，3，4	HZH-4B11-17NNKf	CTGAAATTGATGGAC**NNK**TCCACCCTGAATGGCGACTAC
2	HZH-4B11-18NNKr	GTCGCCATTCAGGGT**MNN**CAGGTCCATCAATTTCAGTGC
2	HZH-4B11-18NNKf	AAATTGATGGACCTG**NNK**ACCCTGAATGGCGACTACACC
2，3	HZH-4B11-20NNKr	TGTAGTCGCCATT**MNN**GGTGGACAGGTCCATCAAT
2，3	HZH-4B11-20NNKf	GGACCTGTCCACC**NNK**AATGGCGACTACACCGACG
2，3，4	HZH-4B11-21NNKr	TGTAGTCGCC**MNN**CAGGGTGGACAGGTCC
2，3，4	HZH-4B11-21NNKf	GTCCACCCTG**NNK**GGCGACTACACCGACG
2	HZH-4B11-22NNKr	CGTCGGTGTAGTC**MNN**ATTCAGGGTGGACAGGTCCA
2	HZH-4B11-22NNKf	TGTCCACCCTGAAT**NNK**GACTACACCGACGAGAAAG
2	HZH-4B11-24NNKr	ACTTTCTCGTCGGT**MNN**GTCGCCATTCAGGGTGGACA
2	HZH-4B11-24NNKf	CCCTGAATGGCGAC**NNK**ACCGACGAGAAAGTAATTGC
2	HZH-4B11-47NNKr	AGCGAGGATAGAT**MNN**GATAGCGGCGGTATTGCCG
2	HZH-4B11-47NNKf	TACCGCCGCTATC**NNK**ATCTATCCTCGCTCTATCC
2	HZH-4B11-52NNKr	GCGAGCAATCGGGAT**MNN**GCGAGGATAGATACTGATAGC
2	HZH-4B11-52NNKf	AGTATCTATCCTCGC**NNK**ATCCCGATTGCTCGCAAAACA
2，3，4	HZH-4B11-73NNKr	GTGTGGGAAGTTGGT**MNN**CGTAGCAATACGGATTTCCGG
2，3，4	HZH-4B11-73NNKf	ATCCGTATTGCTACG**NNK**ACCAACTTCCCACACGGTAAC
2，3，4	HZH-4B11-76NNKr	GTCGTTACCGTGTGG**MNN**GTTGGTTACCGTAGCAATACG

**2，3，4**	HZH-4B11-76NNKf	GCTACGGTAACCAAC**NNK**CCACACGGTAACGACGACATC
**2，3，4**	HZH-4B11-102NNKr	GAACACCAC**MNN**AACTTCATCGGCTCCGTAGGC
**2，3，4**	HZH-4B11-102NNKf	GGAGCCGATGAAGTT**NNK**GTGGTGTTCCCGTAC
**2，3，4**	HZH-4B11-137NNKr	TTTCGATGATCAC**MNN**CAGCAGTACATTCGCTGCC
**2，3，4**	HZH-4B11-137NNKf	GAATGTACTGCTG**NNK**GTGATCATCGAAACCGGCG
**2，3，4**	HZH-4B11-138NNKr	GCCGGTTTCGATGAT**MNN**TTTCAGCAGTACATTCGCTGCC
**2，3，4**	HZH-4B11-138NNKf	GAATGTACTGCTGAAA**NNK**ATCATCGAAACCGGCGAACTG
**2，3，4**	HZH-4B11-139NNKr	TTCGCCGGTTTCGAT**MNN**CACTTTCAGCAGTACATTCGC
**2，3，4**	HZH-4B11-139NNKf	GTACTGCTGAAAGTG**NNK**ATCGAAACCGGCGAACTGAAA
**2，3，4**	HZH-4B11-140NNKr	TCAGTTCGCCGGTTTC**MNN**GATCACTTTCAGCAGTACATTC
**2，3，4**	HZH-4B11-140NNKf	ACTGCTGAAAGTGATC**NNK**GAAACCGGCGAACTGAAAGACG
**2，3，4**	HZH-4B11-141NNKr	TTCAGTTCGCCGGT**MNN**GATGATCACTTTCAGCAGTACA
**2，3，4**	HZH-4B11-141NNKf	GCTGAAAGTGATCATC**NNK**ACCGGCGAACTGAAAGACGA
**2，3，4**	HZH-4B11-165NNKr	GTAGAGGTTTTGAT**MNN**GTCCGCACCCGCTTTGATGG
**2，3，4**	HZH-4B11-165NNKf	AAGCGGGTGCGGAC**NNK**ATCAAAACCTCTACCGGTAA
**2，3，4**	HZH-4B11-166NNKr	ACCGGTAGAGGTTTT**MNN**GAAGTCCGCACCCGCTTTGAT
**2，3，4**	HZH-4B11-166NNKf	GCGGGTGCGGACTTC**NNK**AAAACCTCTACCGGTAAAGTG
**2，3，4**	HZH-4B11-168NNKr	TACCGGTAGA**MNN**TTTGATGAAGTCCGCACCCGCTTTGAT
**2，3，4**	HZH-4B11-168NNKf	GCGGGTGCGGACTTCATCAAA**NNK**TCTACCGGTAAAGTGG
**2，3，4**	HZH-4B11-169NNKr	CCACTTTACCGGT**MNN**GGTTTTGATGAAGTCCGCACC
**2，3，4**	HZH-4B11-169NNKf	GACTTCATCAAAACC**NNK**ACCGGTAAAGTGGCTGTGA
**2，3，4**	HZH-4B11-170NNKr	CACTTTACC**MNN**AGAGGTTTTGATGAAGTCCGCACCCGC
**2，3，4**	HZH-4B11-170NNKf	GCGGACTTCATCAAAACCTCT**NNK**GGTAAAGTGGCTGTG
**2，3，4**	HZH-4B11-171NNKr	GTTCACAGCCACTTT**MNN**GGTAGAGGTTTTGATGAAGTC
**2，3，4**	HZH-4B11-171NNKf	ATCAAAACCTCTACC**NNK**AAAGTGGCTGTGAACGCGACG
**2，3，4**	HZH-4B11-172NNKr	CGTTCACAGCCAC**MNN**ACCGGTAGAGGTTTTGATG
**2，3，4**	HZH-4B11-172NNKf	AACCTCTACCGGT**NNK**GTGGCTGTGAACGCGACGC
**2，3，4**	HZH-4B11-185NNKr	GATCACTTCCAT**MNN**GATGCGCGCGCTTTCCGG
**2，3，4**	HZH-4B11-185NNKf	AGCGCGCGCATC**NNK**ATGGAAGTGATCCGTGAT
**2**	HZH-4B11-197NNKr	ACTTTGAAACCAAC**MNN**TTTTTCTACGCCCATATCACGGATC
**2**	HZH-4B11-197NNKf	TGATATGGGCGTAGAAAAA**NNK**GTTGGTTTCAAAGTGACGGG
**2，3，4**	HZH-4B11-201NNKr	ACGCCGCCCGTCAC**MNN**GAAACCAACGGATTTTTCTA
**2，3，4**	HZH-4B11-201NNKf	AATCCGTTGGTTTC**NNK**GTGACGGGCGGCGTGAGTAC
**2**	HZH-4B11-202NNKr	CCGCCCGT**MNN**TTTGAAACCAACGGATTTTTCTACGCC
**2**	HZH-4B11-202NNKf	GAAAAATCCGTTGGTTTCAAA**NNK**ACGGGCGGCGTGAG

**2**	HZH-4B11-203NNKr	ACTCACGCCGCC**MNN**CACTTTGAAACCAACGGATTTTTC
**2**	HZH-4B11-203NNKf	TCCGTTGGTTTCAAAGTG**NNK**GGCGGCGTGAGTACTGCG
**2，3，4**	HZH-4B11-204NNKr	CAGTACTCACGCC**MNN**CGTCACTTTGAAACCAACG
**2，3，4**	HZH-4B11-204NNKf	TTTCAAAGTGACG**NNK**GGCGTGAGTACTGCGGAAG
**2，3，4**	HZH-4B11-205NNKr	TCCGCAGTACTCAC**MNN**GCCCGTCACTTTGAAACCAA
**2，3，4**	HZH-4B11-205NNKf	TCAAAGTGACGGGC**NNK**GTGAGTACTGCGGAAGATGC
**2**	HZH-4B11-207NNKr	TTCCGCAGT**MNN**CACGCCGCCCGTCAC
**2**	HZH-4B11-207NNKf	GGCGGCGTG**NNK**ACTGCGGAAGATGCG
**2**	HZH-4B11-236NNKr	GGAAGC**MNN**AAAGCGGTAGTGACGCGCATC
**2**	HZH-4B11-236NNKf	CGTCACTACCGCTTT**NNK**GCTTCCGGCCTG
**2，3，4**	HZH-4B11-237NNKr	CCGGA**MNN**ACCAAAGCGGTAGTGACGCGC
**2，3，4**	HZH-4B11-237NNKf	CACTACCGCTTTGGT**NNK**TCCGGCCTGCT
**2，3，4**	HZH-4B11-238NNKr	CAGGCC**MNN**AGCACCAAAGCGGTAGTGACGC
**2，3，4**	HZH-4B11-238NNKf	CTACCGCTTTGGTGCT**NNK**GGCCTGCTGGCA
**2**	HZH-4B11-239NNKr	GCTTGCCAGCAG**MNN**GGAAGCACCAAAGCGGTA
**2**	HZH-4B11-239NNKf	TTTGGTGCTTCC**NNK**CTGCTGGCAAGCCTGTTG
**5**	HZH-2A7-48NNKr	ATAGAGCGAGGATA**MNN**ACTGATAGCGGCGGTATTGC
**5**	HZH-2A7-48NNKf	CCGCCGCTATCAGT**NNK**TATCCTCGCTCTATCCCGAT
**5**	HZH-2A7-49NNKr	GGATAGAGCGAGG**MNN**GATACTGATAGCGGCGGTA
**5**	HZH-2A7-49NNKf	CGCTATCAGTATC**NNK**CCTCGCTCTATCCCGATTG

Note: f = forward primer, r = reverse primer.

3. Screening

The DNA libraries were transformed into chemically-competent *E. coli* BL21 (DE3) cells, and transformants were selected on LB agar plates containing 30 μg/mL kanamycin and lactose (0.2% w/v). After incubation at 37 °C for approximately 16 h, the agar plates were stored at room temperature for 2-3 h. An agar plate-based pre-screening procedure developed earlier[8] was first performed. A heated solution (~ 60 °C) of 0.6 % w/v agarose, 0.5 % v/v DMSO and 0.1 mg/mL 2-hydroxycinnamaldehyde in 10 mM sodium phosphate, pH 7.3 was poured on the agar plates. The plates were incubated for 10 min at room temperature to allow the agarose to solidify. Typically, the colonies producing active enzyme variants were stained red by the probe (2-hydroxycinnamaldehyde).[8] Approximately 890-1300 stained colonies per library were picked with sterile toothpicks, which were used to inoculate 150 μL LB medium supplemented with 30 μg/mL kanamycin and 0.2 % w/v lactose. In each plate, 2 wells were inoculated with a

clean, sterile toothpick (negative control) and 5 wells were inoculated with fresh colonies producing the parental variant for activity reference. The 96-well plates were sealed with sterile gas-permeable seals (Breathe-Easy, Diversified Biotech, Boston, MA, USA) and incubated at 37 °C (180 rpm) overnight. After incubation, cell culture from 96 well plates (50 µL each well) were transferred into plates (96-well Masterblock, Greiner Bio-one, Kremsmünster, Austria) which contained 1 mL LB medium supplemented with 0.2 % w/v lactose, 0.05 % w/v glucose, and 30 µg/mL kanamycin. The 96-deep well plates were sealed with sterile gas-permeable seals and incubated at 37 °C (180 rpm) for 2h, followed by 25 °C (180 rpm) overnight. The bacterial culture was harvested and lysed in 50 µL BugBuster (EMD Biosciences, Madison, WI, USA) containing 0.5 µL/mL benzonase. After 20 min of incubation at room temperature under vigorous shaking, the cell lysates were diluted with 50 µL to 1 mL 20 mM HEPES, 100 mM NaCl, pH 6.5 followed by centrifugation to obtain the cell free extract (CFE). For the screening, 40 µL of the CFE was transferred to 96-well microtiter plate (UV-star F-bottom microplate, Greiner Bio-one, Kremsmünster, Austria) using a robotic pipetting system (JANUS, PerkinElmer). HEPES buffer containing the substrate mixture was then added to each well of the plate to initiate the activity screening. The final reaction mixture (100 µL volume) consisted of the following: 40 % v/v CFE, 0.25 mM **1a**, 20 mM **2**, 5 % (v/v) EtOH and 20 mM HEPES, 100 mM NaCl, pH 6.5. The reaction rate was measured by monitoring the cinnamaldehyde depletion (at 290 nm) at 25 °C in a plate reader (SPECTROstar Omega). The variants with the highest increase in activity compared to the reference were picked from the 96-well plate for plasmid isolation, DNA sequencing and further mutagenesis.

4. Enzyme expression and purification

The plasmid encoding the DERA variant with a C-terminal His-tag was transformed into chemically-competent *E. coli* BL21 (DE3) cells. A single colony was picked to inoculate 5 mL pre-culture (LB medium with 30 µg/mL kanamycin). Typically, following incubation at 37 °C and 200 rpm for 12-16 h, 1 mL pre-culture was used to inoculate 100 mL of LB medium containing kanamycin (30 µg/mL). The cells were incubated at 37 °C, 200 rpm until ~0.5 OD_{600} (2-3 h) was reached, after which the expression was induced by adding 100 µM isopropyl-β-D-thiogalactopyranoside (IPTG), and the culture was further incubated overnight at 25 °C and 200 rpm. After harvesting the cells, the pellet was resuspended in 15 mL 20 mM potassium phosphate, 20 mM imidazole, pH 7, and the cells lysed by ultrasonication, followed by centrifugation to obtain the CFE. The lysate mixture was loaded on a column containing 1 mL

Ni-sepharose and the column washed with 10 mL 20 mM potassium phosphate, 30 mM imidazole, pH 7. The retained protein was eluted with 3 mL 20 mM potassium phosphate, 300 mM imidazole, pH 7. The buffer was exchanged to 20 mM potassium phosphate, pH 7, by using a PD-10 sephadex G-25 gel-filtration column (GE Healthcare, Chicago, IL, USA). The protein concentration was determined by measuring the absorbance at 280 nm with the theoretical molecular weight and extinction coefficient calculated with ProtParam (ExPASy, https://web.expasy.org/protparam/). Purified proteins were analyzed by ESI-MS to confirm the correct molecular mass. The remaining protein solution was snap-frozen in liquid nitrogen and stored at -20 °C until further use.

5. Enzyme activity assay

The DERA peroxygenase activity assays were performed at room temperature by following the decrease in absorbance at 290 nm, which corresponds to the consumption of cinamaldehyde (**1a**). Fresh stock solutions of **1a** (20 mM in EtOH) and **2** (200 mM) were prepared. The final reaction mixture consisted of **1a** (1 mM), **2** (20 mM), and DERA variant (5 µM) in 20 mM HEPES, 100 mM NaCl (pH 6.5) with 5% (v/v) EtOH. For each enzymatic reaction, a negative control reaction without enzyme (enzyme replaced by buffer) was set up in parallel. After the addition of **2**, 300 µL of the reaction mixture was transferred into a 1 mm cuvette for the spectrophotometric analysis. The initial rates (first 10 min) were used for calculating the enzyme activity in Units (U) per mg of protein. 1 U of enzyme activity was defined as the amount of enzyme that catalyzes the conversion of 1 µmol of **1a** per minute (the molar extinction coefficient of **1a** in HEPES buffer at 290 nm was determined to be 23,600 $M^{-1}cm^{-1}$). The initial rates of the enzymatic reactions were corrected by subtracting the rate of the background reaction when no enzyme was present.

6. Substrate scope analysis of DERA-EP

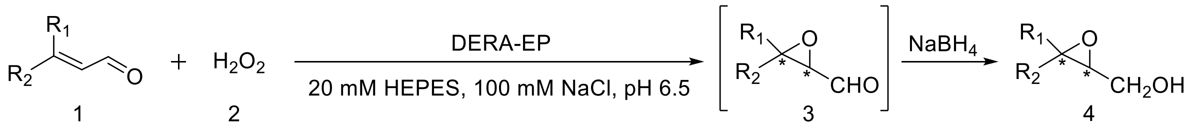

All reactions were performed at room temperature. The reaction mixture (4 mL) consisted of substrate **1** (1 mM), **2** (20 mM), and purified DERA-EP (5 µM) in 20 mM HEPES, 100 mM NaCl (pH 6.5) with 5% (v/v) EtOH (ACN for substrate **1f**, **1g** and **1h**) as cosolvent. For each enzymatic reaction, a negative control reaction without enzyme (enzyme replaced by buffer) was set up in parallel. The reaction was monitored by measuring the depletion of absorbance at the maximum wavelength of the respective aldehyde substrate in a spectrophotometer (Table S2). After completion of the reaction, 200 µL reaction mixture was used for extraction with EtOAc for GC-MS analysis to determine substrate conversion into the desired product. Besides, 1 mL reaction mixture was treated with $NaBH_4$ to reduce product **3** *in situ* to the corresponding alcohol **4**, which was extracted with 500 µL EtOAc and analyzed by chiral HPLC (Figures S14-S27).

Table S2. The maximum wavelength of aldehyde **1** determined by UV-vis analysis.

Compound	Absorption peak (nm)
**1a**	290
**1b**	288
**1c**	284
**1d**	298
**1e**	294
**1f**	300
**1g**	254
**1h**	308
**1i**	284
**1j**	244
**1k**	288

7. Additional potential substrates tested

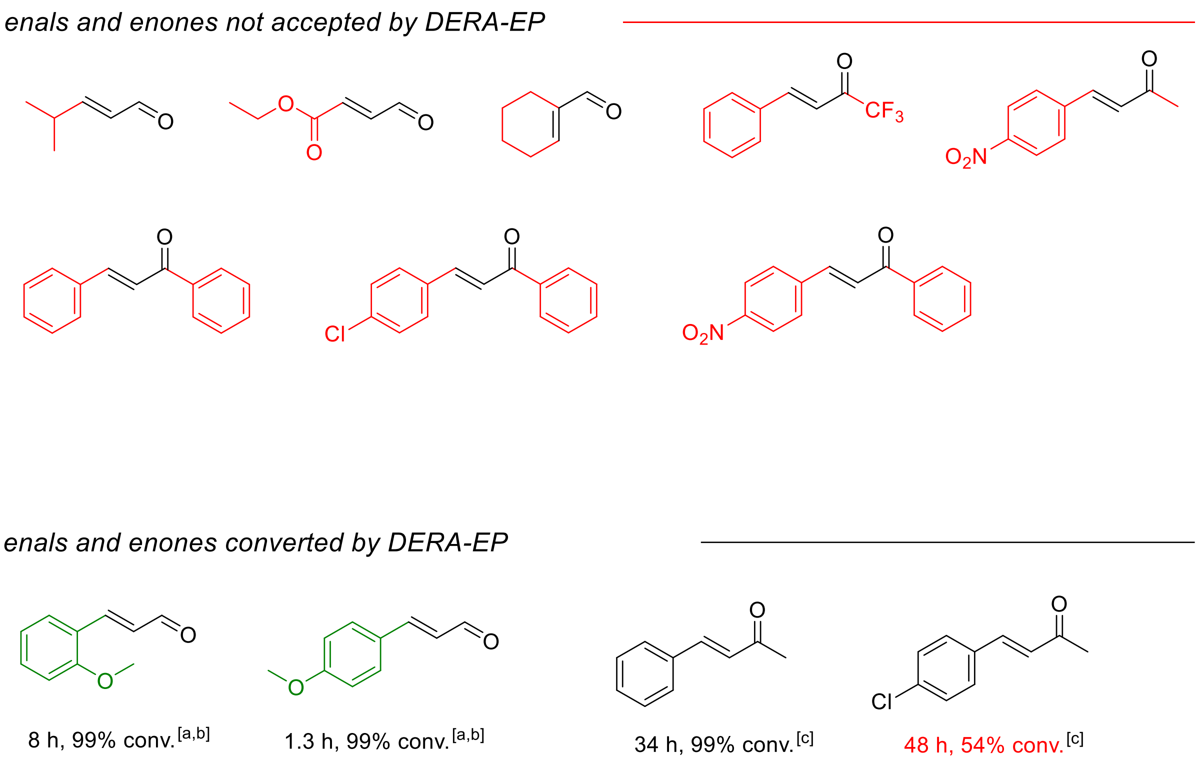

Figure S1. Carbonyl compounds that have been tested as potential substrates for DERA-EP. Reactions were performed with 1 mM *α,β*-unsaturated carbonyl compound, 20 mM **2** in 20 mM HEPES, 100 mM NaCl, pH 6.5, 5% v/v EtOH (10% DMSO for chalcone and chalcone derivatives) at room temperature, reaction volume = 1 mL. Reaction times determined after UV-vis analysis indicated completion of the reaction. Substrate conversion estimated by GC-MS. [a] Reaction with 50 µM DERA-EP. [b] Conversion of *p*-OMe-cinnamaldehyde resulted mainly in *p*-OMe-benzaldehyde rather than the epoxy aldehyde product, whereas conversion of *o*-OMe-cinnamaldehyde resulted in a ~1:3 mixture of *o*-OMe-benzaldehyde and epoxy aldehyde products. [c] Poor substrates that require prolonged incubation with 150 µM DERA-EP.

8. Semi-preparative scale (hundred milligram) synthesis

A reaction mixture was set up containing 5 mM **1a-h and 1j**, 40 mM **2**, 5% (v/v) EtOH (ACN for substrate **1f** and **1g**) and 25 µM of DERA-EP in a final volume of 150 mL in 20 mM HEPES, 100 mM NaCl buffer (pH 6.5). For each reaction, a negative control reaction without enzyme (replaced by buffer) was set up in parallel. The reaction mixture was incubated in a 250 mL Erlenmeyer flask at room temperature, started by adding 5 mM **1** and monitored by following the depletion in absorbance at the maximum wavelength of the aldehyde substrate (Table S2). When the reaction was completed, 400 µL of reaction mixture was sampled for chiral RP-HPLC analysis (reduction by $NaBH_4$, extraction using ethyl acetate followed by evaporation of solvent) to determine the product enantiomeric ratio. Residual H_2O_2 (**2**) in the remaining reaction mixture was removed by the addition of catalase (0.125 mg catalase per mmol H_2O_2) to avoid the formation of peroxyhydrate between the enzymatic epoxy-aldehyde product and H_2O_2.[3,9,10] After 30 min, the mixture was extracted three times with 150 mL ethyl acetate (20 mL pentane for **1j**). The organic layers were combined and washed with 30 mL brine , dried over anhydrous $MgSO_4$, concentrated *in vacuo* evaporation (if ethyl acetate was used) or dried by nitrogen (if pentane was used) followed by filtration, the resulting crude product was (first dried by lyophilization if ethyl acetate was used and) analyzed by NMR to determine the diastereomeric ratio.

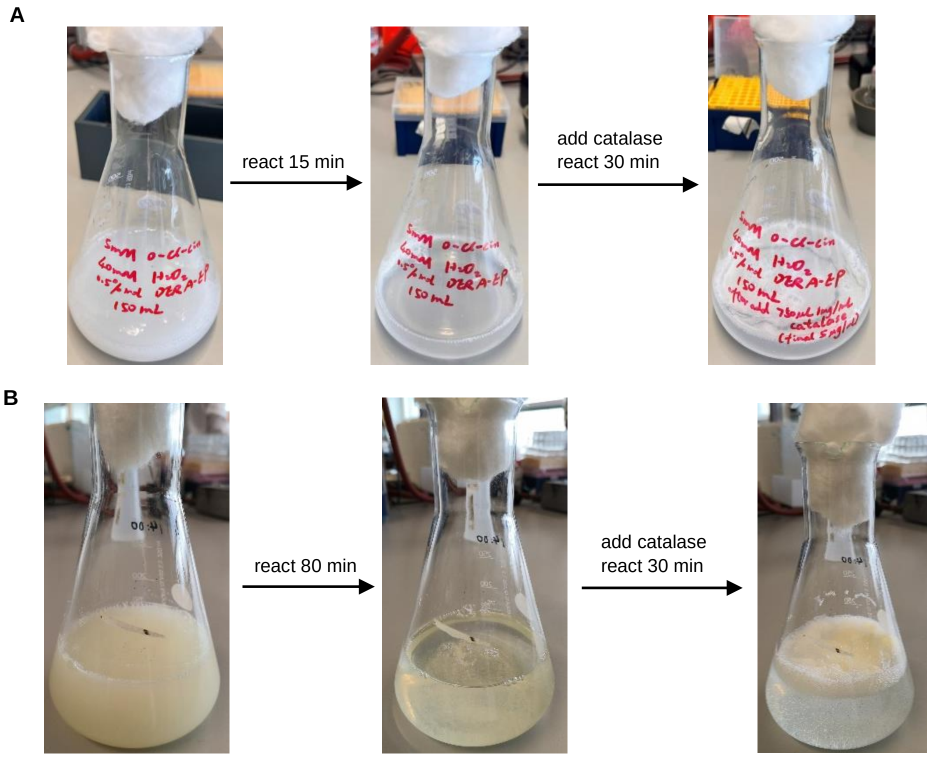

Figure S2. Semi-preparative scale reaction set up and representative alter of reaction mixture. (A) Semi-preparative-scale reaction of **1b**; (B) Semi-preparative-scale reaction of **1h**.

9. Directed evolution overview

Table S3. Overview of the directed evolution of DERA towards enhanced peroxygenase activity.

Round	Input	Evolution strategy	Library size [a]	Output
1	wild-type DERA	Screen DERA variants library [4]	890	9A1, 9E1, 2A1, **4B11**
2	**4B11**	SSM (36 positions, NNK) [b]	1246 (coverage 70%)	**1E1**, 2D12, 14A9, 2A3, 2A8, 2B10, 10A7, 2B9
3	**1E1**	SSM (25 positions, NNK) [c]	890 (coverage 70%)	**2A7**, 2B9, 10A9
4	**2A7**	SSM (24 positions, NNK) [d]	890 (coverage 70%)	5D12, 9F11, 8B8, 8B11, **2A7**
5	5D12, 9F11, 8B8, 8B11, **2A7**	SSM (position 48 and 49, NNK)	1246 (coverage 98%)	11A8 (**DERA-EP**)

[a] Transformants assayed in each round. [b] Residue positions targeted by site-saturation mutagenesis (SSM) in round 2 were: 16, 17, 18, 20, 21, 22, 24, 47, 52, 73, 76, 102, 137, 138, 139, 140, 141, 165, 166, 168, 169, 170, 171, 172, 185, 197, 201, 202, 203, 204, 205, 207, 236, 237, 238, 239. [c] Residue positions targeted by SSM in round 3 were: 16, 17, 20, 21, 73, 76, 102, 137, 138, 139, 140, 141, 165, 166, 168, 169, 170, 171, 172, 185, 201, 204, 205, 237, 238 (the same as targeted position in round 2 except the positions existing in the template DERA 1E1). [d] Residue positions targeted by SSM in round 3 were: 16, 17, 21, 73, 76, 102, 137, 138, 139, 140, 141, 165, 166, 168, 169, 170, 171, 172, 185, 201, 204, 205, 237, 238 (the same as targeted position in round 2 except the positions existing in the template DERA 2A7).

Table S4. Amino acid substitutions compared to wild-type DERA of the DERA variant selected after each round. Amino acid substitutions that newly emerged and are retained in the final variant are highlighted in bold.

Name	Amino acid substitutions compared to wild-type DERA
2A1	T18S, D22G, D24Y, C47S, F52S, T197S, P202V, A203T, S239G
4B11	T18S, D22G, D24Y, C47S, F52S, T197S, P202V, A203T, R207S, S239G
1E1	T18S, D22G, D24Y, C47S, F52S, T197S, P202V, A203T, R207S, **G236S**, S239G
2A7	T18S, **L20S**, D22G, D24Y, C47S, F52S, T197S, P202V, A203T, R207S, G236S, S239G
5D12	T18S, L20S, D22G, D24Y, C47S, F52S, **M185V**, T197S, P202V, A203T, R207S, G236S, S239G
9F11	T18S, L20S, D22G, D24Y, C47S, F52S, **K172R**, T197S, P202V, A203T, R207S, G236S, S239G
8B11	T18S, L20S, D22G, D24Y, C47S, F52S, **V138S**, T197S, P202V, A203T, R207S, G236S, S239G
8B8	T18S, L20S, D22G, D24Y, C47S, F52S, **V138A**, T197S, P202V, A203T, R207S, G236S, S239G
11A8 (DERA-EP)	T18S, L20S, D22G, D24Y, C47S, **I48V**, F52S, **K172R**, T197S, P202V, A203T, R207S, G236S, S239G

10. Proposed mechanism

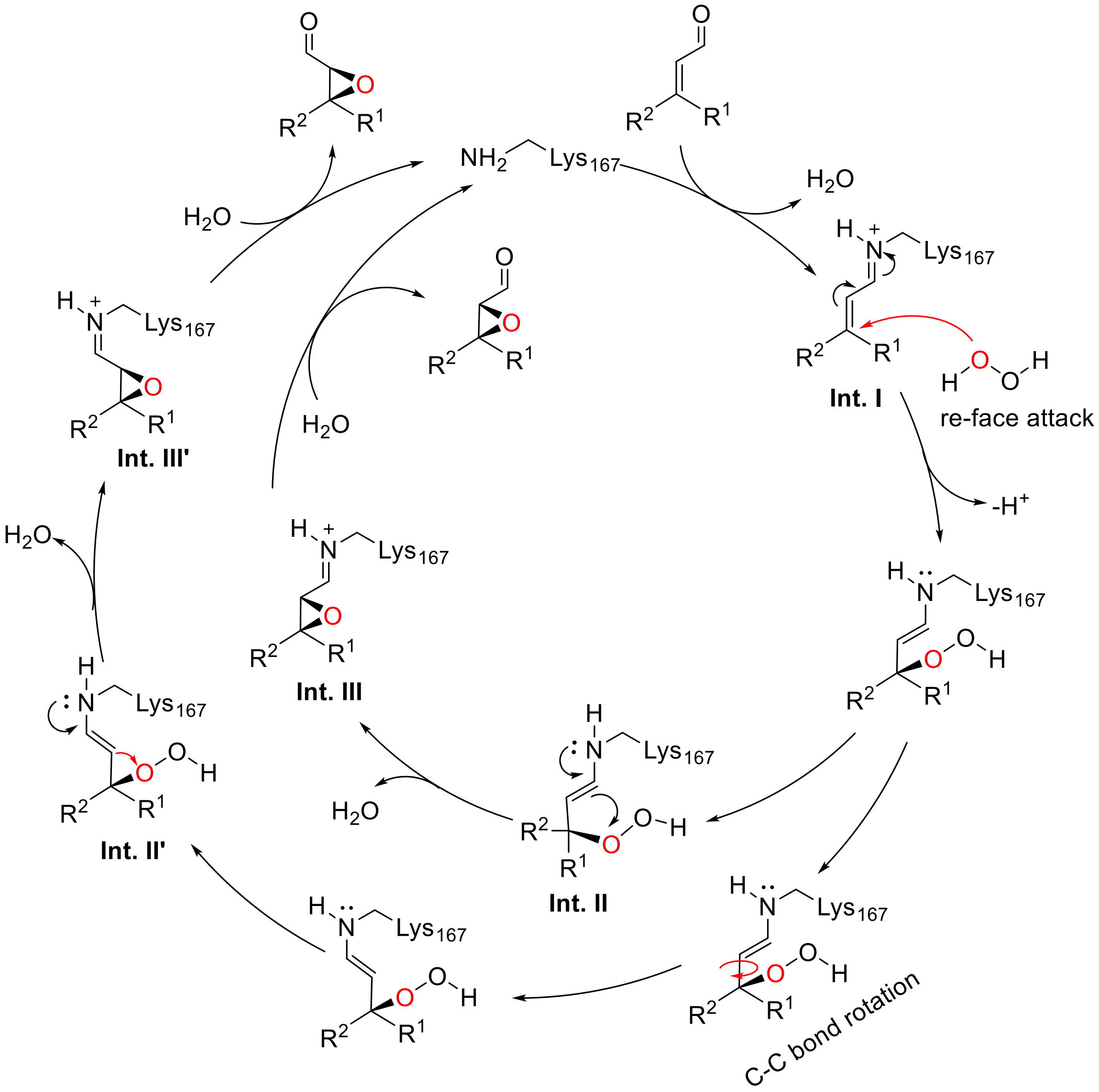

Scheme S1. Proposed catalytic cycle (Int. = intermediate). Based on our knowledge of aldolase[4,5] and iminium-catalyzed epoxidation,[3,11] and the structural studies reported herein, we propose the catalytic active site lysine 167 (ε-amino group) initially reacts with the α,β-unsaturated carbonyl to generate an iminium ion intermediate. Then the nucleophile H_2O_2 exclusively approaches from the *re* face and attacks the electrophilic β-carbon to form the new C3-O bond. Upon ring closure and subsequent hydrolysis of the epoxy iminium ion, the chiral epoxide is formed. The *anti* product presumably is formed by rotation of the C2-C3 bond after the formation of C3-O bond, but before ring closure.

11. X-ray crystallography

Screening for initial crystallization conditions was carried out using the sitting-drop vapour-diffusion method at 20 °C in MRC 96-well 2-drop crystallization plates (Molecular Dimensions) with crystallization solutions from the JCSG-plus, TOP96 and PACT screens (all purchased from Molecular

Dimensions). Drops of 200 nL were prepared by mixing purified DERA-EP (8 mg/mL in 20 mM potassium phosphate, pH 7) with reservoir solutions at two volume ratio's (75:125 and 125:75) with the help of a Mosquito pipetting system (SPT Labtech). The best crystals were obtained with 10 % (v/v) 2-propanol, 20 % (w/v) PEG 4000 in 0.1 M HEPES and 25 % (w/v) PEG 3350 in 0.1 M HEPES, pH 7.5 (conditions a6 and c7, respectively, from the TOP96 screen). Subsequent optimization was performed by screening against gradients of isopropanol and PEG 3350 in 0.1 M HEPES, pH 7.5. Crystals suitable for crystallography grew in a few days from solutions containing 0-4% 2-propanol, 22-24% PEG 3350 in 0.1 M HEPES, pH 7.5. Crystals were cryoprotected in reservoir solution containing 25% (v/v) glycerol and flash-cooled in liquid nitrogen. To trap a Schiff base reaction intermediate, several crystals were briefly soaked (about 30 seconds up to 1 minute) in cryoprotection solutions containing saturating amounts of 2-chloro-cinnamaldehyde, 3-chloro-cinnamaldehyde, 4-chloro-cinnamaldehyde or 4-nitro-cinnamaldehyde. X-ray diffraction data were collected at the ID30A-1 (MASSIF-1) beamline of the European Synchroton Radition Facility (ESRF), Grenoble and processed with the program XDS[12] and with the AIMLESS routines[13] from the CCP4 software suite[14] Crystals diffracted to a maximum resolution ranging between 1.4 and 1.8 Å, belonged to space group $P2_1$ and contained two polypeptide chains in the asymmetric unit with a solvent content of ~39%. Initial phases for apo DERA-EP (maximum resolution 1.4 Å) were obtained by molecular replacement with the program Phaser[15] using the crystal structure of DERA-MA (PDB entry 7P75) as a search model. The apo DERA-EP model was improved by several rounds of model building and refinement, using the programs Coot[16] and REFMAC5,[17] respectively. The final rounds included the placement and validation of waters and glycerol molecules and the refinement of anisotropic atomic B-factors. Of the substrate-soaked crystals, only the ones treated with 4-chloro-cinnamaldehyde and 4-nitro-cinnamaldehyde allowed observation of additional electron density extending from the side chain of Lys167, indicative of the formation of a substrate-derived enzyme-Schiff-base intermediate. Refinement of the DERA-EP complexes with bound 4-chloro-cinnamaldehyde and 4-nitro-cinnamaldehyde was carried out at 1.52 Å and 1.58 Å resolution, respectively, with REFMAC5, using similar protocols as for apo DERA-EP. Geometry dictionary files for the lysine-linked substrates were calculated with the CCP4 AceDRG program.[18] Validation of the final structures was performed with Molprobity[19] and the wwPDB Validation Server at https://validate.wwpdb.org. PyMOL (Schrödinger) was used for structure analysis and figure preparation. Coordinates and structure factors were deposited at the PDB with entry codes 9FD7 (apo DERA-EP), 9FD8 (DERA-EP/4-chloro-CIN) and 9FD9 (DERA-EP/4-nitro-CIN). A summary of the data collection and refinement statistics are available in Table S5.

Table S5. Crystallographic statistics data collection and refinement DERA-EP.

	apo	4-chloro-CIN	4-nitro-CIN
**Data collection**			
Beamline	ESRF ID30A-1	ESRF ID30A-1	ESRF ID30A-1
Wavelength (Å)	0.9655	0.9655	0.9655
Space group	$P2_1$	$P2_1$	$P2_1$
Unit cell dimensions			
a,b,c, (Å)	48.2, 72.8, 70.9	48.1, 72.5, 70.9	48.1, 72.5, 70.7
β (°)	96.2	96.2	96.3
Resolution range (Å)	73-1.40 (1.42-1.40)*	48-1.58 (1.61-1.58)	48-1.52 (1.55-1.52)
Total observations	604885 (20006)	208588 (6512)	164586 (8355)
Unique reflections	91423 (3626)	65329 (2935)	70796 (3451)
Completeness (%)	95.7 (76.5)	98.7 (89.6)	95.5 (93.6)
Multiplicity	6.6 (5.5)	3.2 (2.2)	2.3 (2.4)
$\langle I/\sigma \rangle$	16.9 (2.2)	7.2 (1.0)	7.0 (0.8)
$CC_{(1/2)}$	0.999 (0.746)	0.996 (0.455)	0.988 (0.312)
R_{meas}	0.064 (0.875)	0.101 (0.862)	0.115 (1.471)
R_{pim}	0.025 (0.354)	0.055 (0.542)	0.072 (0.900)
**Refinement**			
R-factor/R_{free} #	0.134/0.157	0.164/0.185	0.186/0.217
Number of non-H atoms			
protein	3699	3684	3713
ligands, water	30, 208	26, 240	30, 247
Average B-factors (Å^2)			
protein	17.1	18.6	20.2
ligands, water	35.5, 33.3	20.9, 31.8	30.5, 31.3
RMSD			
bond lengths (Å)	0.0109	0.0096	0.0109
bond angles (°)	1.7	1.6	1.8
Ramachandran plot			
%favoured, outliers	98.3, 0.0	98.3, 0.0	98.3, 0.0
Rotamers, %outliers	1.0	0.8	1.2
Clashscore	1.19	1.87	2.77
**PDB entry**	9FD7	9FD8	9FD9

* Values in parentheses correspond to highest resolution shell.

# Rfree is calculated as R-factor using 5% of all reflections randomly chosen, which were excluded from structure refinement.

12. DNA and Protein Sequences

> DNA sequence of DERA 5’→3’

ATGACTGATCTGAAAGCAAGCAGCCTGCGTGCACTGAAATTGATGGACCTGACCACCCTG
AATGACGACGACACCGACGAGAAAGTAATTGCTCTGTGTCATCAGGCCAAAACCCCGGT
CGGCAATACCGCCGCTATCTGTATCTATCCTCGCTTTATCCCGATTGCTCGCAAAACACTG
AAAGAGCAGGGCACCCCGGAAATCCGTATTGCTACGGTAACCAACTTCCCACACGGTAA
CGATGACATCGAAATCGCGCTGGCAGAAACCCGTGCGGCAATCGCCTACGGTGCCGATG
AAGTTGACGTGGTGTTCCCGTACCGCGCGCTGATGGCGGGTAACGAGCAGGTTGGTTTTG
ACCTGGTGAAAGCCTGTAAAGAGGCTTGCGCGGCAGCGAATGTACTGCTGAAAGTGATC
ATCGAAACCGGCGAACTGAAAGACGAAGCGCTGATCCGTAAAGCGTCTGAAATCTCCAT
CAAAGCGGGTGCGGACTTCATCAAAACCTCTACCGGTAAAGTGGCTGTGAACGCGACGC
CGGAAAGCGCGCGCATCATGATGGAAGTGATCCGTGATATGGGCGTAGAAAAAACCGTT
GGTTTCAAACCAGCGGGCGGCGTGCGTACTGCGGAAGATGCGCAGAAATATCTCGCCATC
GCAGATGAACTGTTCGGTGCTGACTGGGCAGATGCGCGTCACTACCGCTTTGGTGCTTCC
AGCCTGCTGGCAAGCCTGTTGAAAGCGCTGGGCCACGGTGACGGTAAGAGCGCCAGCAG
CTACCTCGAGCACCACCACCACCACCACTGA

> protein sequence of DERA wild-type

MTDLKASSLRALKLMDLTTLNDDDTDEKVIALCHQAKTPVGNTAAICIYPRFIPIARKTLKEQ
GTPEIRIATVTNFPHGNDDIEIALAETRAAIAYGADEVDVVFPYRALMAGNEQVGFDLVKACK
EACAAANVLLKVIIETGELKDEALIRKASEISIKAGADFIKTSTGKVAVNATPESARIMMEVIR
DMGVEKTVGFKPAGGVRTAEDAQKYLAIADELFGADWADARHYRFGASSLLASLLKALGH
GDGKSASSYLEHHHHHH

> DNA sequence of DERA-EP 5’→3’

ATGACTGATCTGAAAGCAAGCAGCCTGCGTGCACTGAAATTGATGGACCTGTCCACCTCT
AATGGCGACTACACCGACGAGAAAGTAATTGCTCTGTGTCATCAGGCCAAAACCCCGGTC
GGCAATACCGCCGCTATCAGTGTTTATCCTCGCTCTATCCCGATTGCTCGCAAAACACTGA
AAGAGCAGGGCACCCCGGAAATCCGTATTGCTACGGTAACCAACTTCCCACACGGTAAC
GACGACATCGAAATCGCGCTGGCAGAAACCCGTGCGGCAATCGCCTACGGAGCCGATGA
AGTTGACGTGGTGTTCCCGTACCGCGCGCTGATGGCGGGTAACGAGCAGGTTGGTTTTGA
CCTGGTGAAAGCTTGTAAAGAGGCCTGCGCGGCAGCGAATGTACTGCTGAAAGTGATCAT
CGAAACCGGCGAACTGAAAGACGAAGCGCTGATCCGTAAAGCGTCTGAAATCTCCATCA
AAGCGGGTGCGGACTTCATCAAAACCTCTACCGGTAGGGTGGCTGTGAACGCGACGCCG
GAAAGCGCGCGCATCATGATGGAAGTGATCCGTGATATGGGCGTAGAAAAATCCGTTGG
TTTCAAAGTGACGGGCGGCGTGAGTACTGCGGAAGATGCGCAGAAATATCTCGCCATCGC
AGATGAGCTGTTCGGTGCTGACTGGGCAGATGCGCGTCACTACCGCTTTAGTGCTTCCGG
CCTGCTGGCAAGCCTGTTGAAAGCGCTGGGCCACGGTGATGGTAAGAGCGCCAGCAGCT
ACCTCGAGCACCACCACCACCACCACTGA

> protein sequence of DERA-EP

MTDLKASSLRALKLMDLSTSNGDYTDEKVIALCHQAKTPVGNTAAISVYPRSIPIARKTLKEQ
GTPEIRIATVTNFPHGNDDIEIALAETRAAIAYGADEVDVVFPYRALMAGNEQVGFDLVKACK
EACAAANVLLKVIIETGELKDEALIRKASEISIKAGADFIKTSTGRVAVNATPESARIMMEVIRD
MGVEKSVGFKVTGGVSTAEDAQKYLAIADELFGADWADARHYRFSASGLLASLLKALGHG
DGKSASSYLEHHHHHH

Figure S3. DNA and protein sequence of DERA wild-type and DERA-EP, the mutated amino acids are shown in red.

13. NMR Spectra

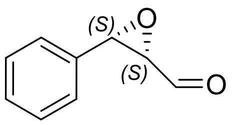

major isomer
(*anti*)

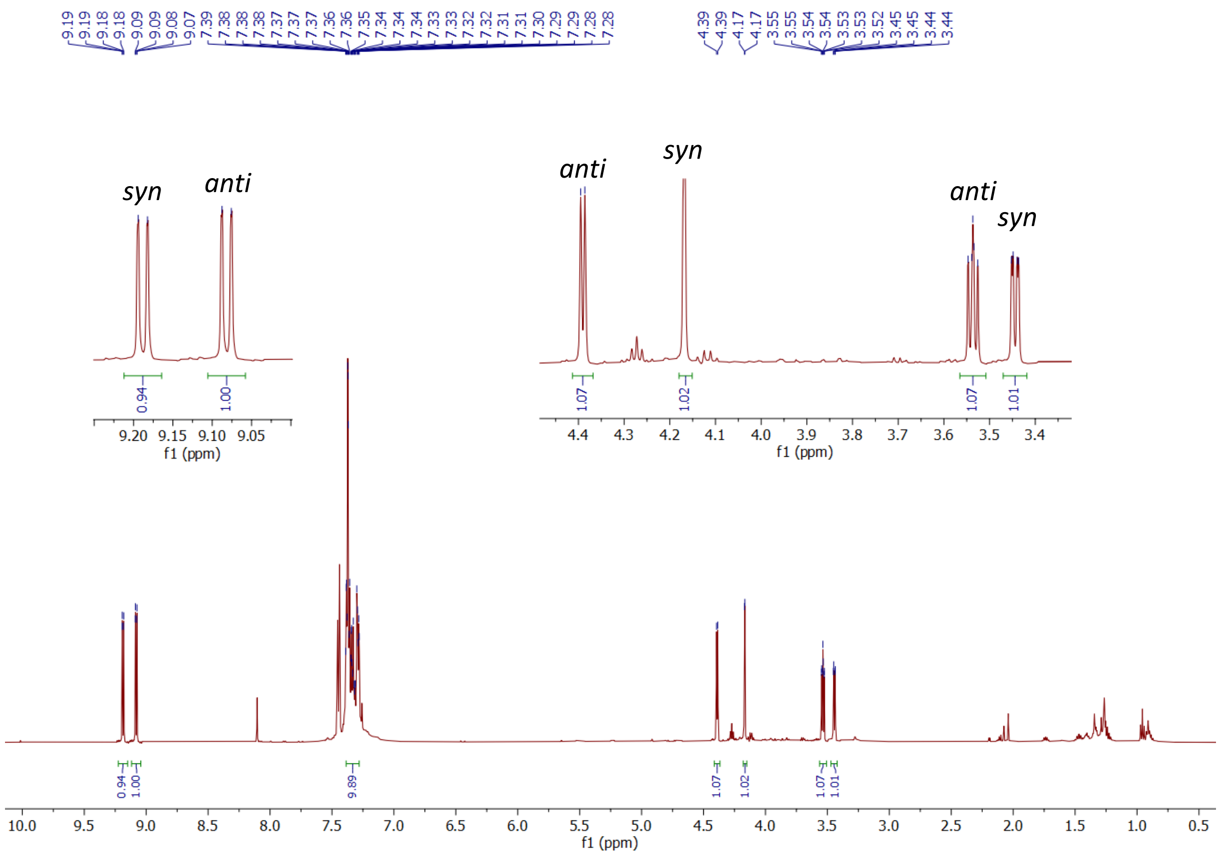

Figure S4. ^{1}H-NMR of 3-phenyloxirane-2-carbaldehyde (**3a**) obtained by semi-preparative-scale synthesis using DERA-EP. Colorless oil. Major diastereomer: *anti*-(2*S*, 3*S*). ^{1}H NMR (500 MHz, $CDCl_3$, *anti* diastereomer) δ 9.08 (dd, *J* = 6.0, 0.8 Hz, 1H), 7.39 – 7.28 (m, 5H), 4.39 (d, *J* = 4.5 Hz, 1H), 3.54 (ddd, *J* = 5.8, 4.6, 0.8 Hz, 1H). ^{1}H NMR (500 MHz, $CDCl_3$, *syn* diastereomer) δ 9.19 (dd, *J* = 6.0, 0.8 Hz, 1H), 7.39 – 7.28 (m, 5H), 4.17 (d, *J* = 1.8 Hz, 1H), 3.44 (dd, *J* = 6.2, 1.8 Hz, 1H). ^{1}H NMR data is in agreement with literature.[9,11,20,21]

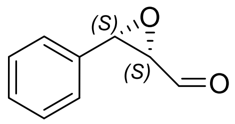

major isomer
(*anti*)

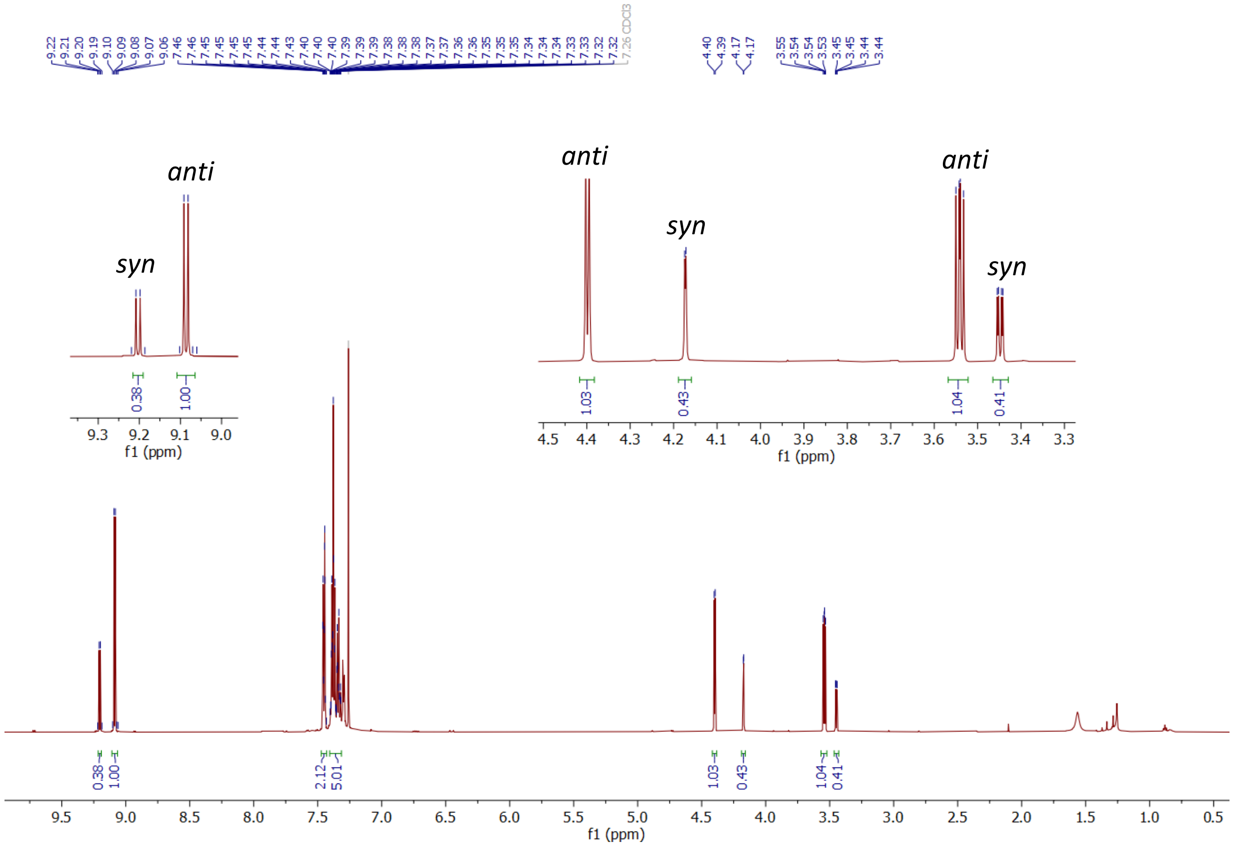

Figure S5. ^{1}H-NMR of 3-phenyloxirane-2-carbaldehyde (**3a**) obtained by semi-preparative-scale synthesis using DERA-2A1. Colorless oil. Major diastereomer: *anti*-(2*S*, 3*S*). ^{1}H NMR (600 MHz, $CDCl_3$, *anti* diastereomer) δ 9.09 (dd, *J* = 6.0, 1H), 7.41 – 7.31 (m, 5H), 4.40 (d, *J* = 4.6 Hz, 1H), 3.54 (dd, *J* = 6.1, 4.6 Hz, 1H). ^{1}H NMR (600 MHz, $CDCl_3$, *syn* diastereomer) δ 9.20 (dd, *J* = 6.0 Hz, 1H), 7.48 – 7.43 (m, 5H), 4.17 (d, *J* = 1.8 Hz, 1H), 3.45 (dd, *J* = 6.0, 1.8 Hz, 1H). ^{1}H NMR data is in agreement with literature.[9,11,20,21]

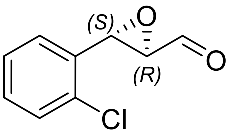

major isomer
(*syn*)

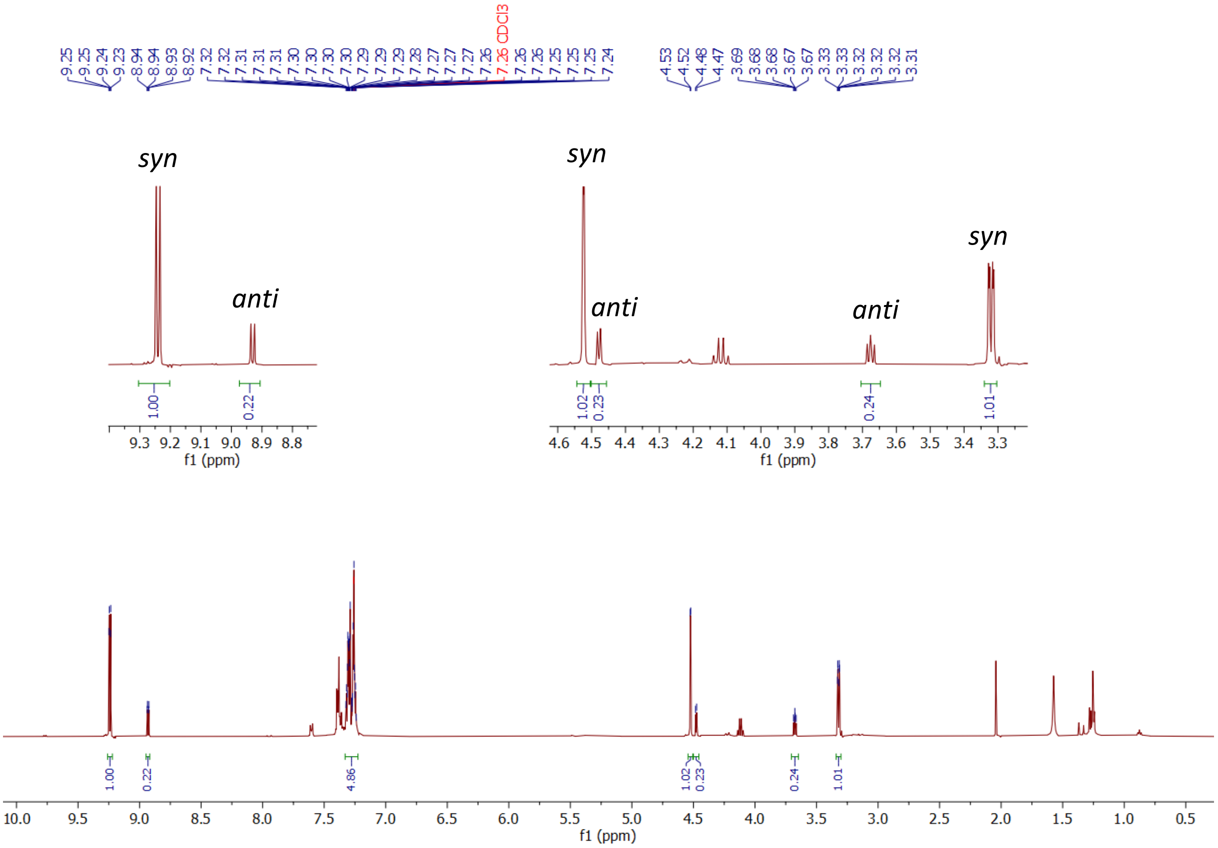

Figure S6. ^{1}H-NMR of 3-(2-chlorophenyl)oxirane-2-carbaldehyde (**3b**) obtained by semi-preparative-scale synthesis using DERA-EP. Yellow oil. Major diastereomer: *syn*-(2*R*, 3*S*). ^{1}H NMR (500 MHz, $CDCl_3$, *anti* diastereomer) δ 8.93 (d, J = 6.0 Hz, 1H), 7.32 – 7.24 (m, 4H), 4.48 (d, J = 4.6 Hz, 1H), 3.70 – 3.65 (m, 1H). ^{1}H NMR (500 MHz, $CDCl_3$, *syn* diastereomer) δ 9.24 (dd, J = 6.0, 0.9 Hz, 1H), 7.32 – 7.24 (m, 4H), 4.52 (d, J = 1.8 Hz, 1H), 3.32 (dt, J = 6.4, 1.3 Hz, 1H). ^{1}H NMR data is in agreement with literature.[9]

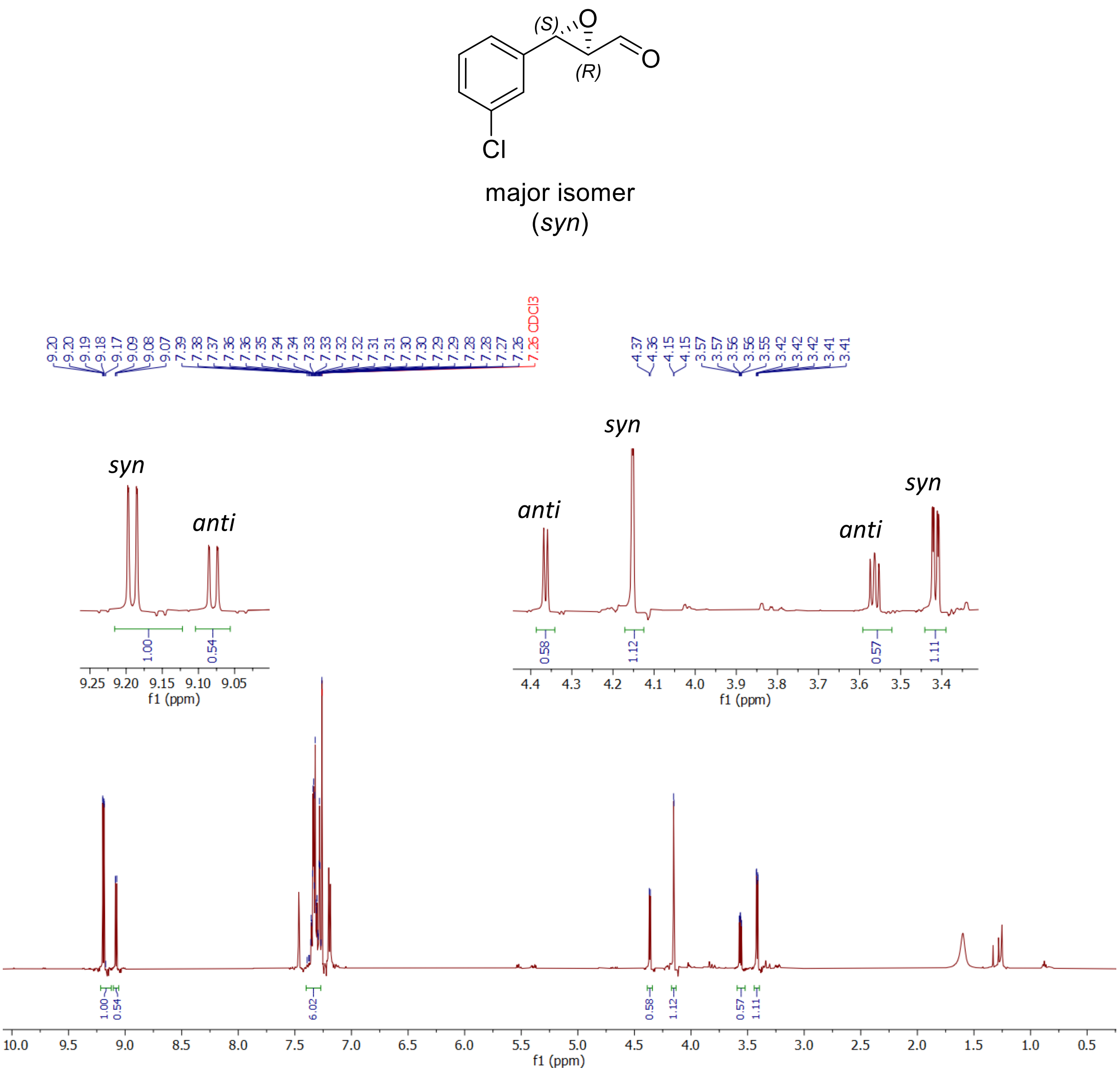

Figure S7. ^{1}H-NMR of 3-(3-chlorophenyl)oxirane-2-carbaldehyde (**3c**) obtained by semi-preparative-scale synthesis using DERA-EP. Yellow oil. Major diastereomer: *syn*-(2*R*, 3*S*). ^{1}H NMR (500 MHz, $CDCl_3$, *anti* diastereomer) δ 9.10 – 9.06 (m, 1H), 7.40 – 7.27 (m, 4H), 4.36 (d, *J* = 4.6 Hz, 1H), 3.59 – 3.52 (m, 1H). ^{1}H NMR (500 MHz, $CDCl_3$, *syn* diastereomer) δ 9.19 (dd, *J* = 6.0, 0.7 Hz, 1H), 7.40 – 7.27 (m, 4H), 4.15 (d, *J* = 1.8 Hz, 1H), 3.44 – 3.40 (m, 1H). ^{1}H NMR data is in agreement with literature.[9]

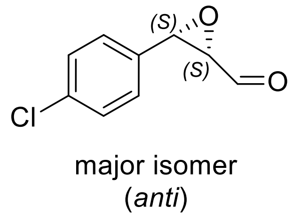

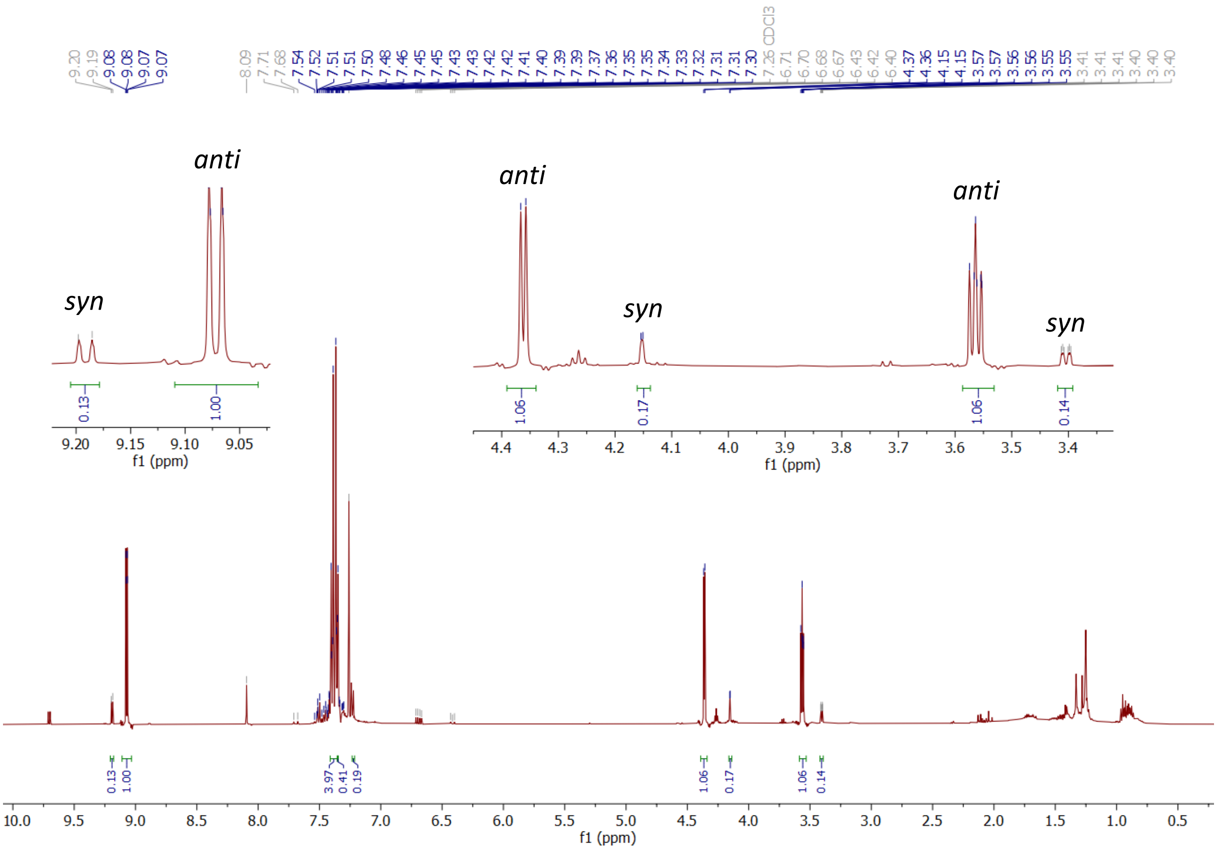

Figure S8. ^{1}H-NMR of 3-(4-chlorophenyl)oxirane-2-carbaldehyde (**3d**) obtained by semi-preparative-scale synthesis using DERA-EP. Yellow oil. Major diastereomer: *anti*-(2*S*, 3*S*). ^{1}H NMR (500 MHz, $CDCl_3$, *anti* diastereomer) δ 9.11 – 9.04 (m, 1H), 7.41 – 7.36 (m, 4H), 4.36 (d, *J* = 4.6 Hz, 1H), 3.57 – 3.55 (m, 1H). ^{1}H NMR (500 MHz, $CDCl_3$, *syn* diastereomer) δ 9.19 (d, *J* = 6.3 Hz, 1H), 7.35 (s, 2H), 7.22 (s, 2H), 4.15 (d, *J* = 1.8 Hz, 1H), 3.40 (d, *J* = 5.9 Hz, 1H). ^{1}H NMR data is in agreement with literature.[11]

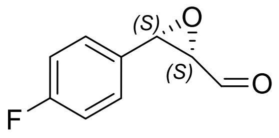

major isomer
(*anti*)

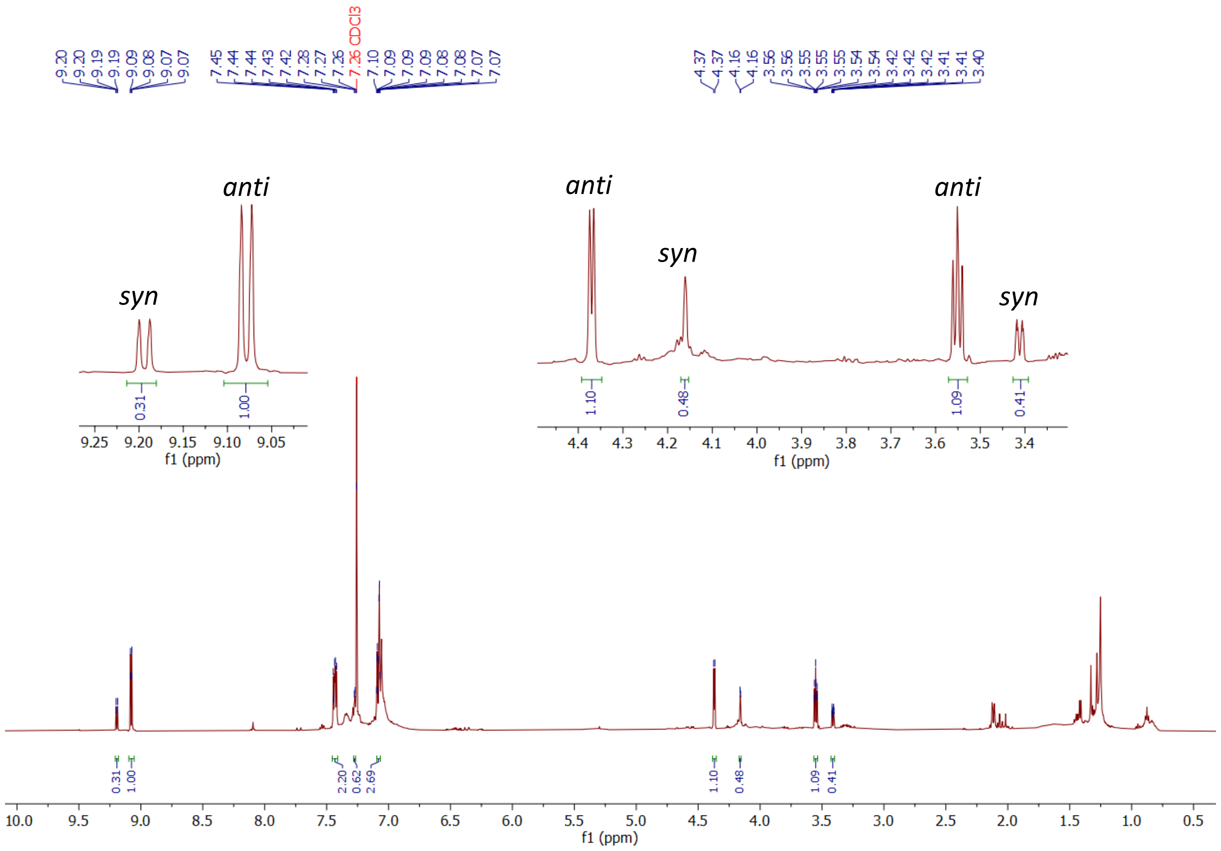

Figure S9. ^{1}H-NMR of 3-(3-fluorophenyl)oxirane-2-carbaldehyde (**3e**) obtained by semi-preparative-scale synthesis using DERA-EP. Yellow oil. Major diastereomer: *anti*-(2*S*, 3*S*). ^{1}H NMR (500 MHz, $CDCl_3$, *anti* diastereomer) δ 9.08 (dd, *J* = 5.8, 1.1 Hz, 1H), 7.43 (dd, *J* = 8.4, 5.2 Hz, 2H), 7.08 (dd, *J* = 8.6, 1.3 Hz, 2H), 4.37 (d, *J* = 4.5 Hz, 1H), 3.55 (dd, *J* = 5.9, 4.6 Hz, 1H). ^{1}H NMR (500 MHz, $CDCl_3$, *syn* diastereomer) δ 9.19 (dd, *J* = 6.0, 1.1 Hz, 1H), 7.27 (d, *J* = 3.2 Hz, 2H), 7.08 (dd, *J* = 8.6, 1.3 Hz, 2H), 4.16 (d, *J* = 1.8 Hz, 1H), 3.41 (dt, *J* = 5.9, 1.5 Hz, 1H). ^{1}H NMR data is in agreement with literature.[9,20]

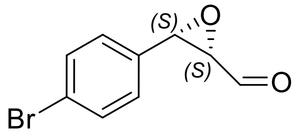

major isomer
(*anti*)

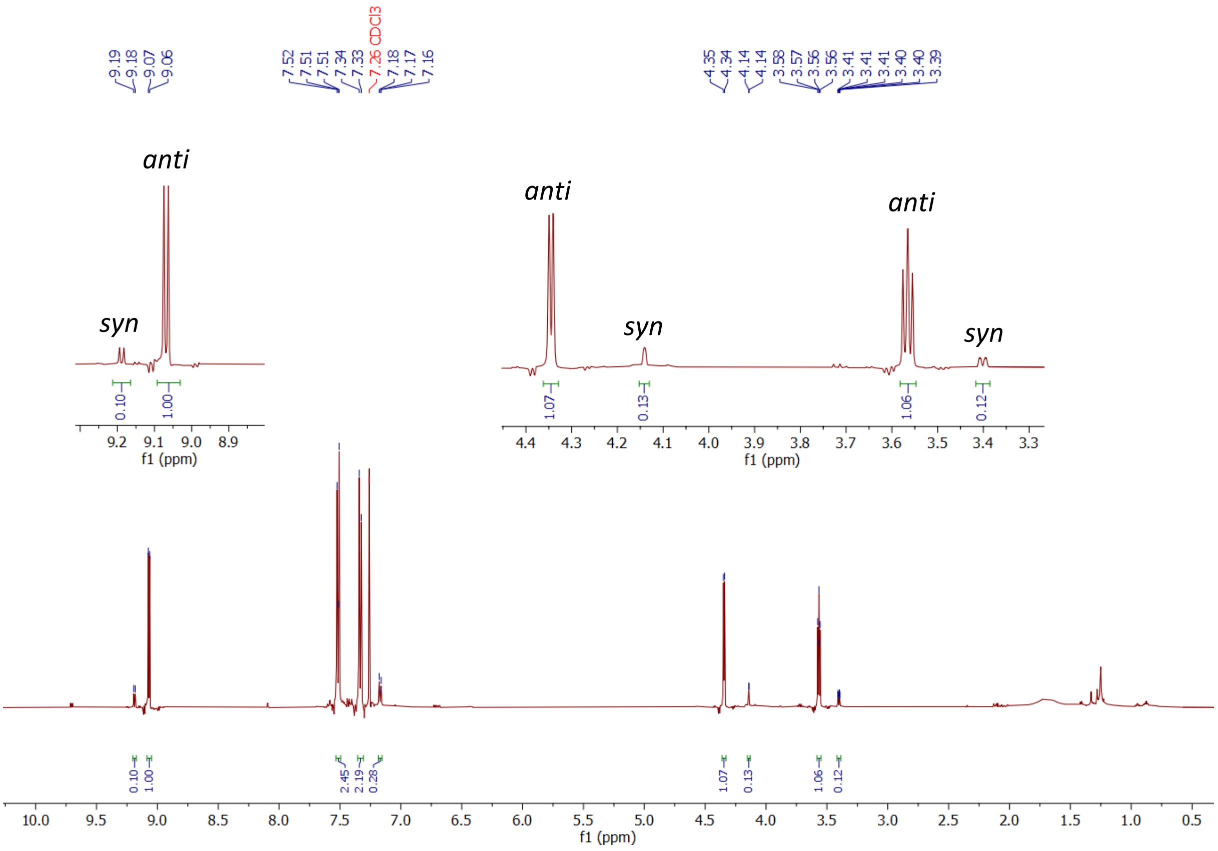

Figure S10. ^{1}H-NMR of 3-(4-bromophenyl)oxirane-2-carbaldehyde (**3f**) obtained by semi-preparative-scale synthesis using DERA-EP. Yellow oil. Major diastereomer: *anti*-(2*S*, 3*S*). ^{1}H NMR (500 MHz, $CDCl_3$, *anti* diastereomer) δ 9.07 (d, *J* = 5.8 Hz, 1H), 7.52 (d, *J* = 8.3 Hz, 2H), 7.33 (d, *J* = 8.2 Hz, 2H), 4.34 (d, *J* = 4.5 Hz, 1H), 3.58 – 3.55 (m, 1H). ^{1}H NMR (500 MHz, $CDCl_3$, *syn* diastereomer) δ 9.19 (d, *J* = 6.0 Hz, 1H), 7.52 (d, *J* = 8.3 Hz, 2H), 7.17 (d, *J* = 8.2 Hz, 2H), 4.14 (d, *J* = 1.8 Hz, 1H), 3.40 (dd, *J* = 5.9 Hz, 1H). ^{1}H NMR data is in agreement with literature.[9,20]

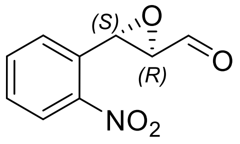

major isomer
(*syn*)

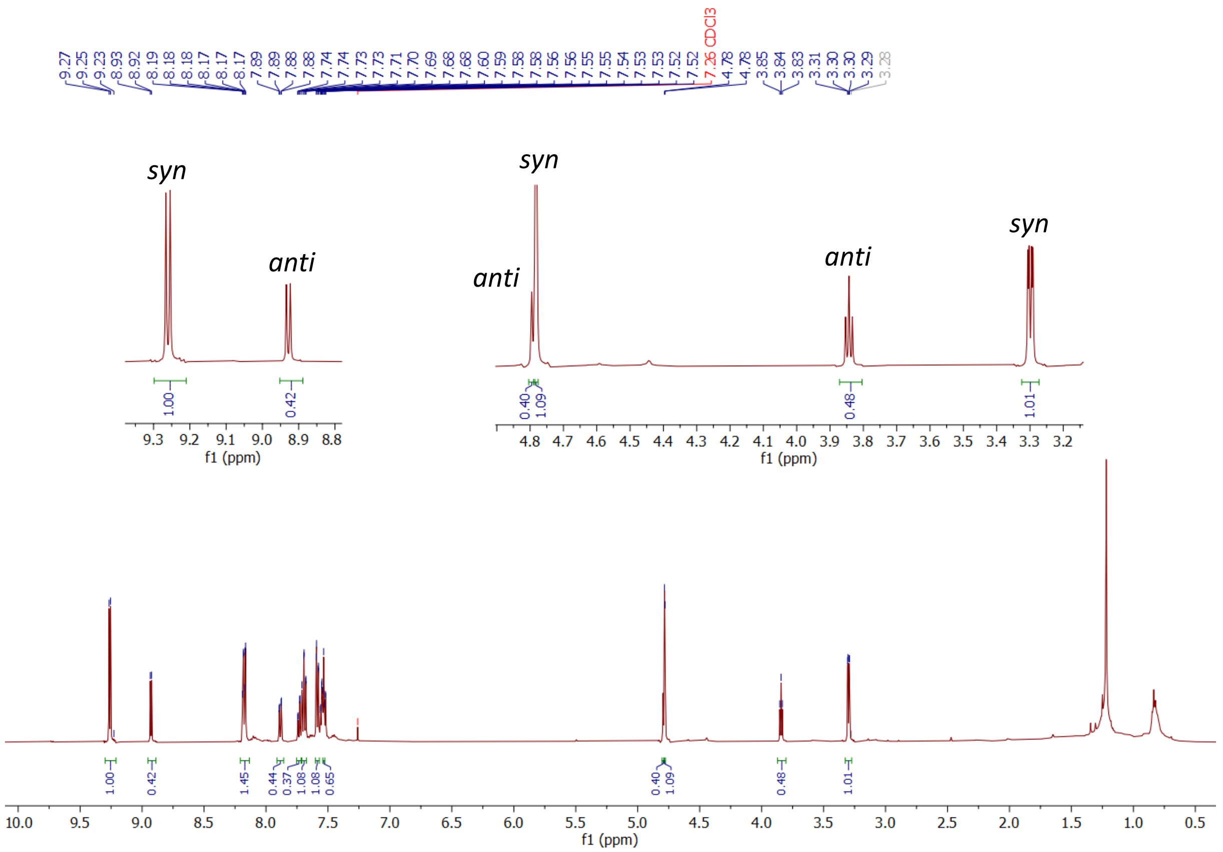

Figure S11. ^{1}H-NMR of 3-(2-nitrophenyl)oxirane-2-carbaldehyde (**3g**) obtained by semi-preparative-scale synthesis using DERA-EP. Yellow oil. Major diastereomer: *syn*-(2*R*, 3*S*). ^{1}H NMR (500 MHz, $CDCl_3$, *anti* diastereomer) δ 8.93 (d, J = 5.3 Hz, 1H), 8.18 (dt, J = 8.2, 2.0 Hz, 1H), 7.88 (dd, J = 7.8, 1.4 Hz, 1H), 7.73 (dd, J = 7.6, 1.2 Hz, 1H), 7.53 (d, J = 3.0 Hz, 1H), 4.80 (s, 1H), 3.84 (t, J = 5.1 Hz, 1H). ^{1}H NMR (500 MHz, $CDCl_3$, *syn* diastereomer) δ 9.26 (d, J = 5.8 Hz, 1H), 8.18 (dt, J = 8.2, 2.0 Hz, 1H), 7.88 (dd, J = 7.8, 1.4 Hz, 1H), 7.69 (dd, J = 7.6, 1.2 Hz, 1H), 7.59 (dd, J = 7.9, 1.5 Hz, 1H), 4.78 (d, J = 2.2 Hz, 1H), 3.30 (dd, J = 5.8, 2.0 Hz, 1H). ^{1}H NMR data is in agreement with literature.[11]

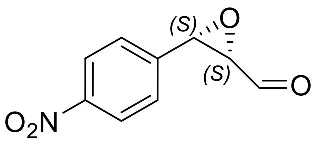

major isomer
(*anti*)

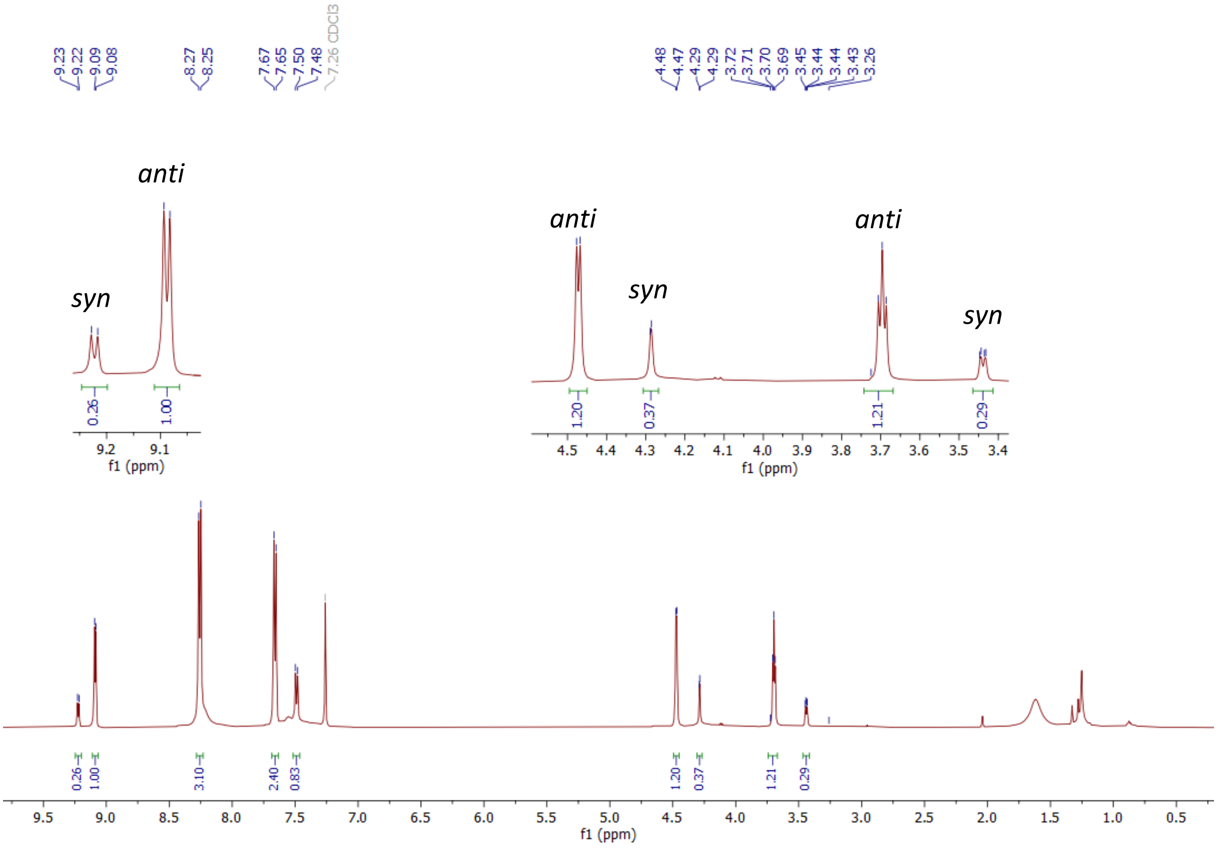

Figure S12. ^{1}H-NMR of 3-(4-nitrophenyl)oxirane-2-carbaldehyde (**3h**) obtained by semi-preparative-scale synthesis using DERA-EP. Yellow oil. Major diastereomer: *anti*-(2*S*, 3*S*). ^{1}H NMR (500 MHz, $CDCl_3$, *anti* diastereomer) δ 9.09 (d, *J* = 5.5 Hz, 1H), 8.26 (dd, *J* = 8.4 Hz, 2.0 Hz, 2H), 7.66 (d, *J* = 8.3 Hz, 2H), 4.47 (d, *J* = 4.6 Hz, 1H), 3.70 (t, *J* = 5.1 Hz, 1H). ^{1}H NMR (500 MHz, $CDCl_3$, *syn* diastereomer) δ 9.22 (d, *J* = 5.9 Hz, 1H), 8.26 (dd, *J* = 8.4, 2.0 Hz, 2H), 7.66 (d, *J* = 8.3 Hz, 1H), 7.49 (d, *J* = 8.6 Hz, 1H), 4.31-4.27 (m, 1H), 3.44 (dd, *J* = 6.0, 1.7 Hz, 1H). ^{1}H NMR data is in agreement with literature.[20]

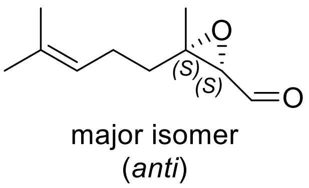

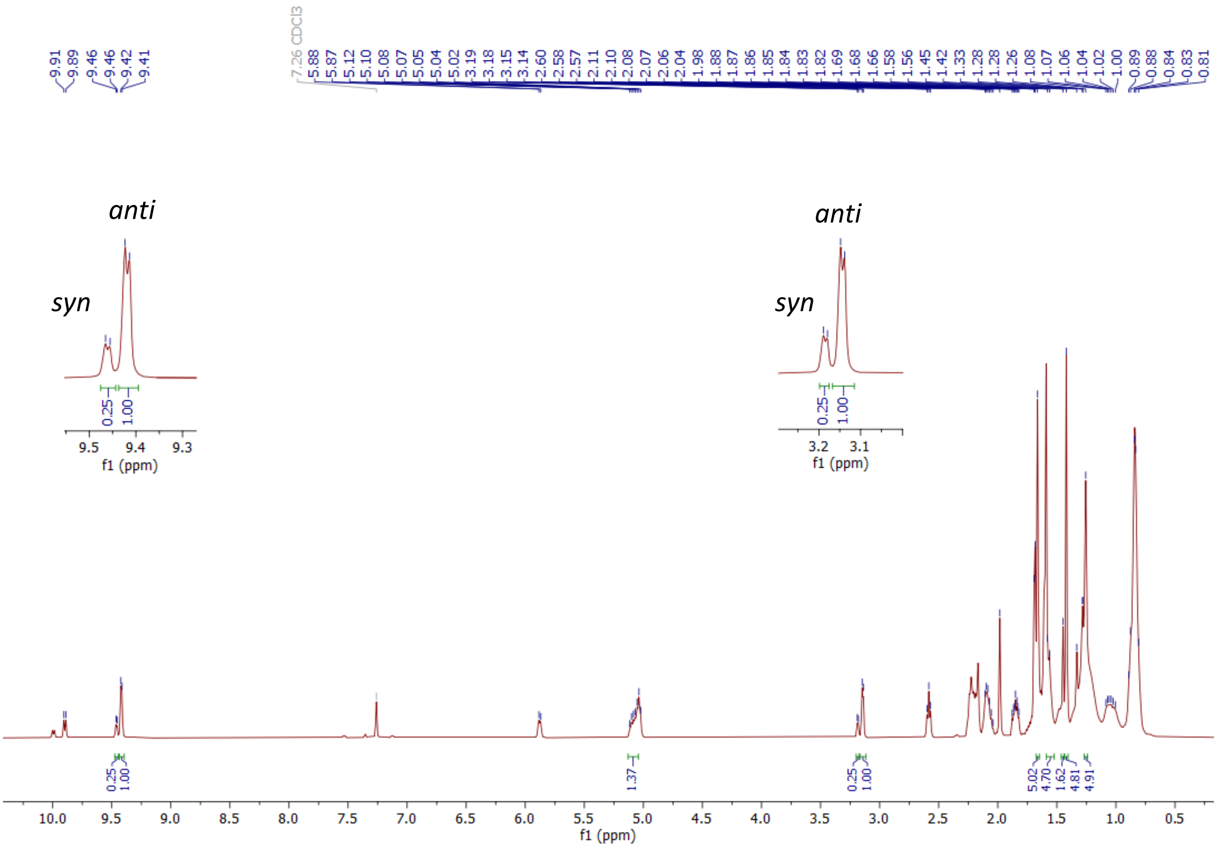

Figure S13. ^{1}H-NMR of 3-methyl-3-(4-methylpent-3-en-1-yl)oxirane-2-carbaldehyde (**3j**) obtained by semi-preparative-scale synthesis using DERA-EP. Transparent oil. Major diastereomer: *anti*-(2*S*, 3*S*). ^{1}H NMR (500 MHz, $CDCl_3$, *anti* diastereomer) δ 9.42 (d, *J* = 5.0 Hz, 1H), 5.15 – 5.05 (m, 1H), 3.16 (dd, J = 20.7, 4.9 Hz, 1H), 1.66 (s, 3H), 1.57 (d, J = 9.2 Hz, 3H), 1.45 (s, 1H), 1.42 (s, 3H), 1.26 (s, 3H). ^{1}H NMR (500 MHz, $CDCl_3$, *syn* diastereomer) δ 9.46 (d, *J* = 4.8 Hz, 1H), 5.15 – 5.05 (m, 1H), 3.16 (dd, J = 20.7, 4.9 Hz, 1H), 1.66 (s, 3H), 1.57 (d, J = 9.2 Hz, 3H), 1.45 (s, 1H), 1.42 (s, 3H), 1.26 (s, 3H). ^{1}H NMR data is in agreement with literature.[11,20]

14. Normal-phase Chiral HPLC Analysis

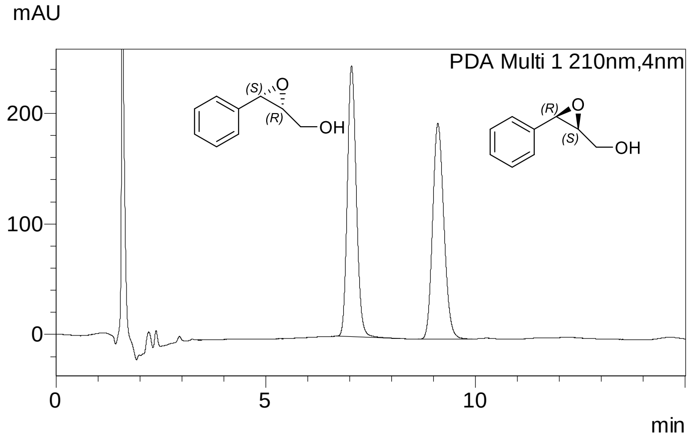

PDA Ch1 210nm			
Peak#	Ret. Time	Area	Area%
1	7,050	3649115	49,916
2	9,110	3661338	50,084
Total		7310453	100,000

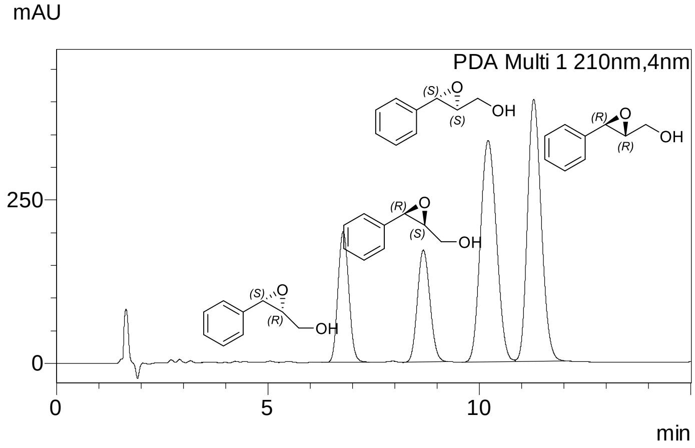

PDA Ch1 210nm			
Peak#	Ret. Time	Area	Area%
1	6,784	3550133	14,121
2	8,680	3529906	14,041
3	10,207	8961392	35,646
4	11,292	9098808	36,192
Total		25140240	100,000

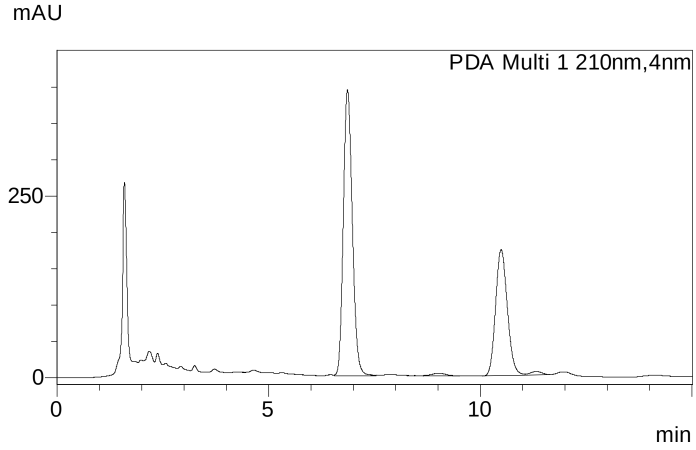

PDA Ch1 210nm			
Peak#	Ret. Time	Area	Area%
1	6,866	5799621	62,192
2	9,018	79645	0,854
3	10,494	3354026	35,967
4	11,323	92033	0,987
Total		9325325	100,000

Figure S14. Chiral normal-phase HPLC chromatogram of product **4a**. From top to bottom are as follows: racemic *anti* product (purified from a chemically synthesized mixture of racemic *syn*- and *anti*-**3a** by using a silica gel column using EtOAc/pentane); chemically synthesized racemic product (*syn* and *anti* mixture); and enzymatic product synthesized with DERA-EP. Epoxy-aldehyde was reduced by $NaBH_4$ to the corresponding alcohol for analysis. HPLC method: CHIRALCEL® OD-H column (150 mm × 4.6 mm, column temperature = 25°C), Heptane/Isopropanol = 95:5, flow rate = 1.2 mL/min, λ = 210 nm. Retention time: *anti*-(2*R*, 3*S*) = 6.8 min, *anti*-(2*S*,3*R*) = 8.7 min, *syn*-(2*S*, 3*S*) = 10.2 min, *syn*-(2*R*, 3*R*) =11.3 min. The

assignment of the absolute configuration of enzymatic product was based on previously reported chiral normal-phase HPLC data.[22]

15. Reverse-phase Chiral HPLC Analysis

Table S6. Reverse-phase Chiral HPLC method[3] and results summary.

Entry	Compound	Column[b]	Mobile phase[c]	Retention time[d] (min)				
				anti-1	*syn*-(2*S*, 3*S*)	*syn*-(2*R*, 3*R*)	*anti*-2	Substrate **1**
1	**4a**	AD-RH	90% H_2O, 10% ACN	31.2 (2*R*, 3*S*)	34.8	48.5	61.5 (2*S*, 3*R*)	83.6
2	**4b**	AD-RH	80% H_2O, 20% ACN	26.4	22.9	28.9	63.1	40.3
3	**4c**	AD-RH	80% H_2O, 20% ACN	22.1	27.1	50.3	73.1	61.1
4	**4d**	ID	82% H_2O, 18% ACN	18.6	20.2	29.4	18.6	33.2
5	**4e**	AD-RH	90% H_2O, 10% ACN	57.5	49.8	63.0	57.5	117.6
6	**4f**	ID	82% H_2O, 18% ACN	26.2	29.7	49.6	27.6	48.8
7	**4g**	AD-RH	60% H_2O, 40% ACN	4.6	6.2	8.9	15.9	4.2
8	**4h**	AD-RH	80% H_2O, 20% ACN	30.5	23.7	43.5	30.6	46.7
9	**4i**	ID	80% H_2O, 20% ACN	8.5	9.6	13.1	12.1	14.6
10	**4j**	AD-RH	80% H_2O, 20% ACN	19.1	20.4	28.0	51.6	69.3 & 95.2
11	**4k**	AD-RH	70% H_2O, 30% ACN	4.8	5.3	13.0	16.2	8.7

[a] UV detection at 210 nm. [b] Column information: Daicel CHIRALPAK® AD-RH (150 × 4.6 mm, 5 µm) and CHIRALPAK® ID (150 × 4.6 mm, 5 µm); column temperature = 25°C. [c] Isocratic; flow = 1 mL/min. [d] Numbers in the tables are from racemic product and authentic or substrate samples.

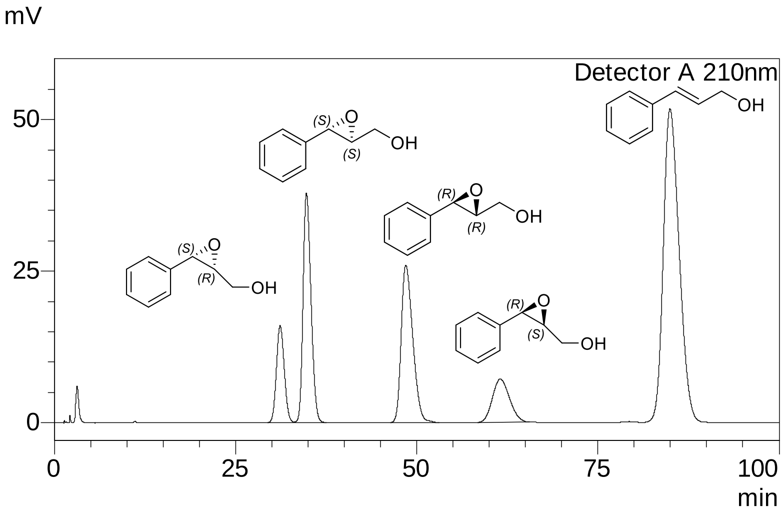

Detector A 210nm

Peak#	Ret. Time	Area	Area%
1	31,159	1210704	14,848
2	34,788	2877419	35,289
3	48,502	2884209	35,372
4	61,546	1181590	14,491
Total		8153921	100,000

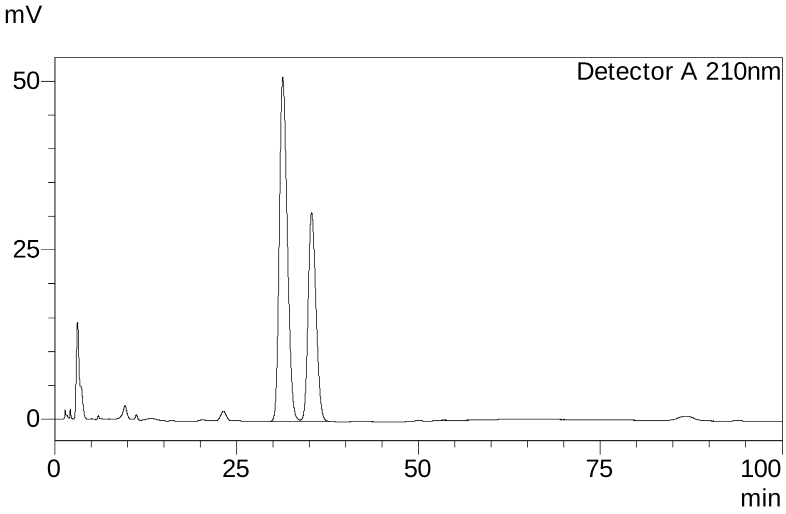

Detector A 210nm

Peak#	Ret. Time	Area	Area%
1	31,361	3891381	62,533
2	35,324	2331553	37,467
Total		6222933	100,000

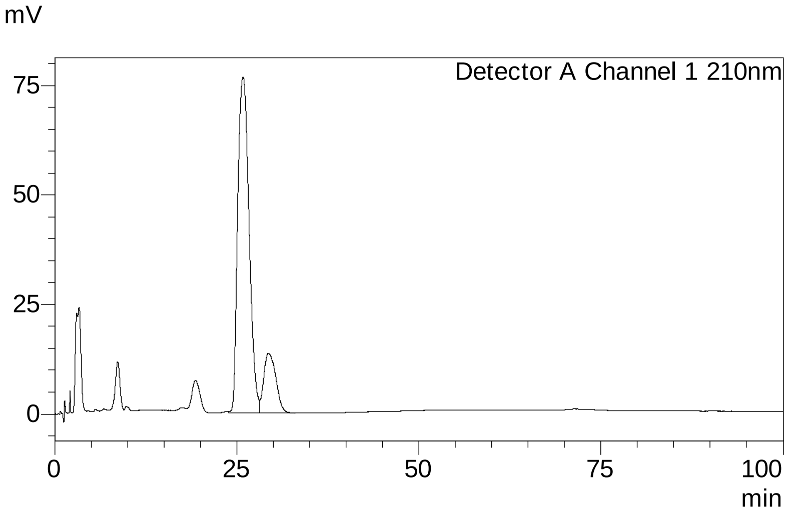

Detector A Channel 1 210nm

Peak#	Ret. Time	Area	Area%
1	25,874	8316476	84,016
2	29,367	1582237	15,984
Total		9898714	100,000

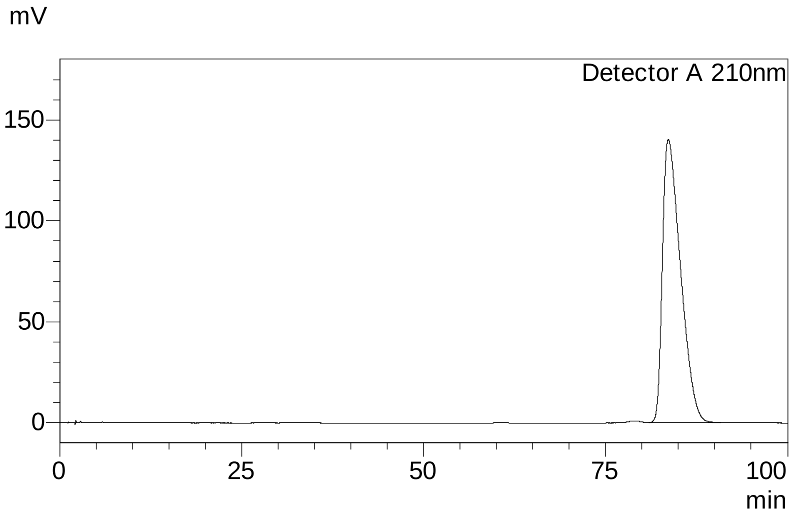

Detector A 210nm

Peak#	Ret. Time	Area	Area%
1	83,626	24236023	100,000
Total		24236023	100,000

Figure S15. Chiral reverse-phase HPLC chromatogram of product **4a**. From top to bottom are as follows: chemically synthesized racemic product and authentic cinnamyl alcohol; enzymatic product generated with DERA-EP using H_2O_2 as oxidant; enzymatic product generated with DERA-EP using *t*-BuOOH as oxidant; and control reaction (without enzyme). The assignment of the absolute configuration of enzymatic product was based on previously reported chiral reverse-phase HPLC data.[3]

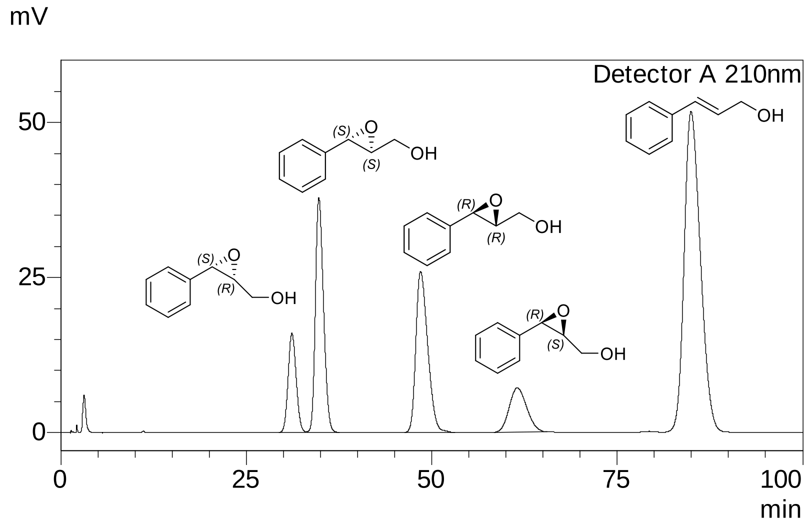

Detector A 210nm

Peak#	Ret. Time	Area	Area%
1	31,159	1210704	14,848
2	34,788	2877419	35,289
3	48,502	2884209	35,372
4	61,546	1181590	14,491
Total		8153921	100,000

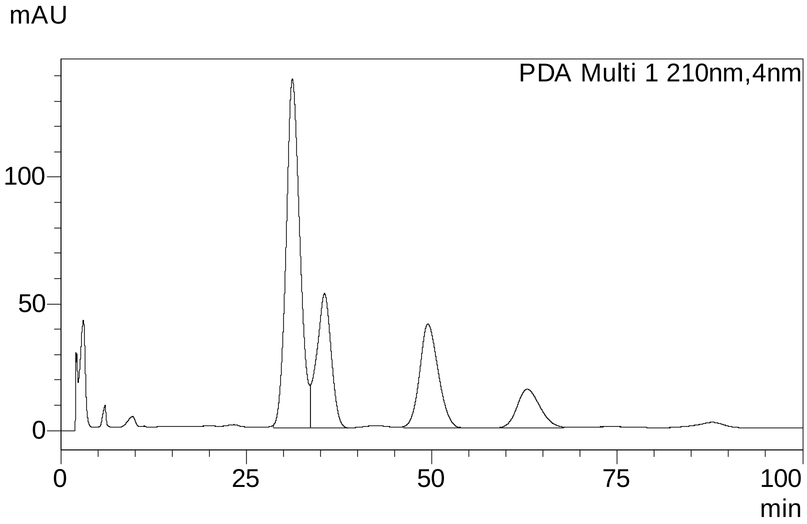

PDA Ch1 210nm

Peak#	Ret. Time	Area	Area%
1	31,214	17814582	50,031
2	35,555	7283653	20,456
3	49,473	7168095	20,131
4	62,853	3340560	9,382
Total		35606891	100,000

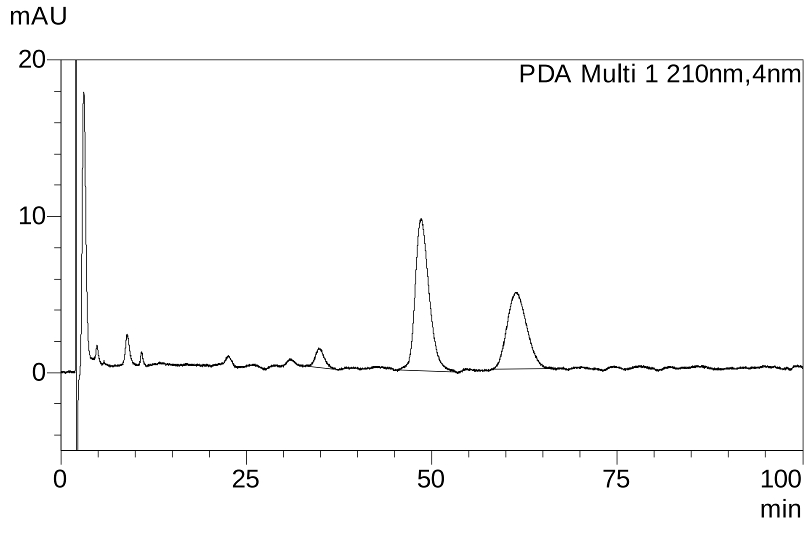

PDA Ch1 210nm

Peak#	Ret. Time	Area	Area%
1	34,856	106052	4,633
2	48,573	1270437	55,495
3	61,342	912783	39,872
Total		2289272	100,000

Figure S16. Chiral reverse-phase HPLC chromatogram of product **4a**. From top to bottom are as follows: chemically synthesized racemic product and authentic cinnamyl alcohol; racemic product spiked with enzymatic product generated with DERA-EP; and enzymatic product synthesized by 4-OT YIA. The assignment of the absolute configuration of enzymatic product was based on previously reported chiral reverse-phase HPLC data.[3]

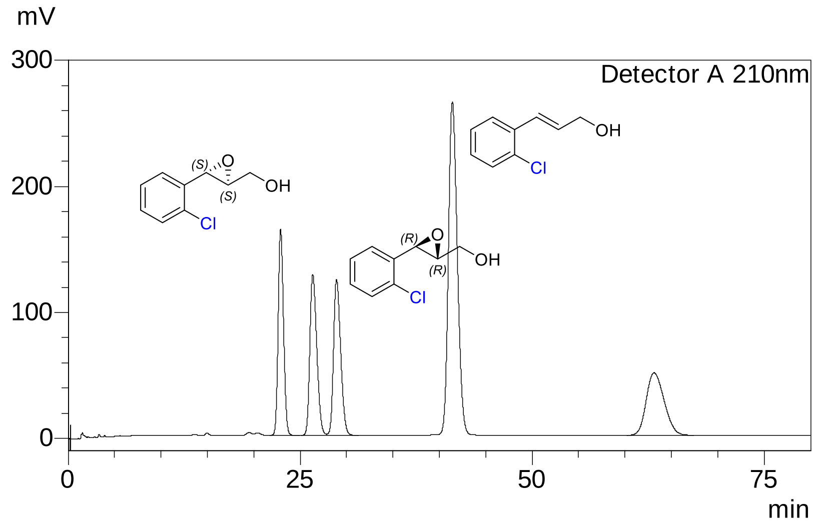

Detector A 210nm

Peak#	Ret. Time	Area	Area%
1	22,892	6567981	24,855
2	26,360	6631315	25,094
3	28,922	6596516	24,963
4	63,140	6629707	25,088
Total		26425519	100,000

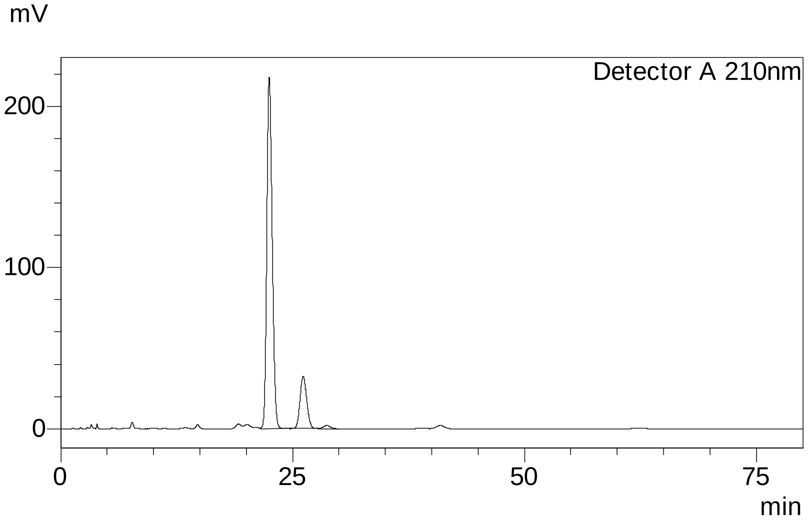

Detector A 210nm

Peak#	Ret. Time	Area	Area%
1	22,472	8836451	83,577
2	26,139	1633111	15,446
3	28,686	103263	0,977
Total		10572825	100,000

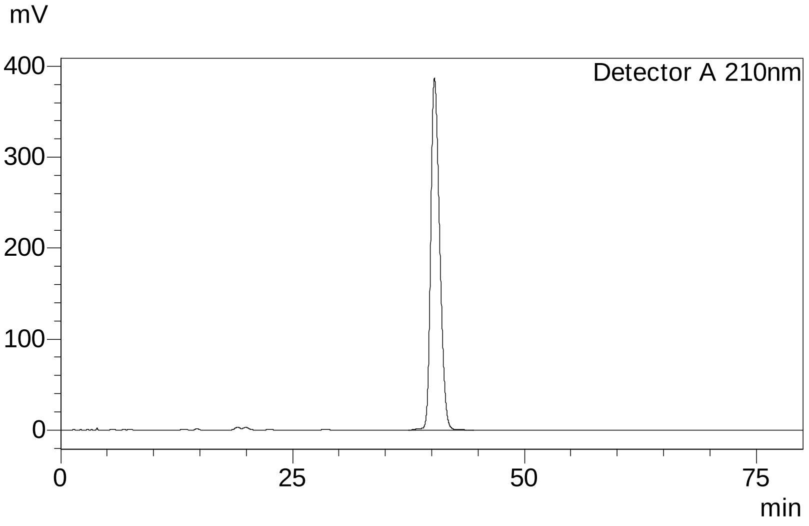

Detector A 210nm

Peak#	Ret. Time	Area	Area%
1	40,292	25979684	100,000
Total		25979684	100,000

Figure S17. Chiral reverse-phase HPLC chromatogram of product **4b**. From top to bottom are as follows: chemically synthesized racemic product and authentic (*E*)-3-(2-chlorophenyl)prop-2-en-1-ol; enzymatic product synthesized with DERA-EP; and control reaction (without enzyme). The assignment of the absolute configuration of enzymatic product was based on previously reported chiral reverse-phase HPLC data.[3]

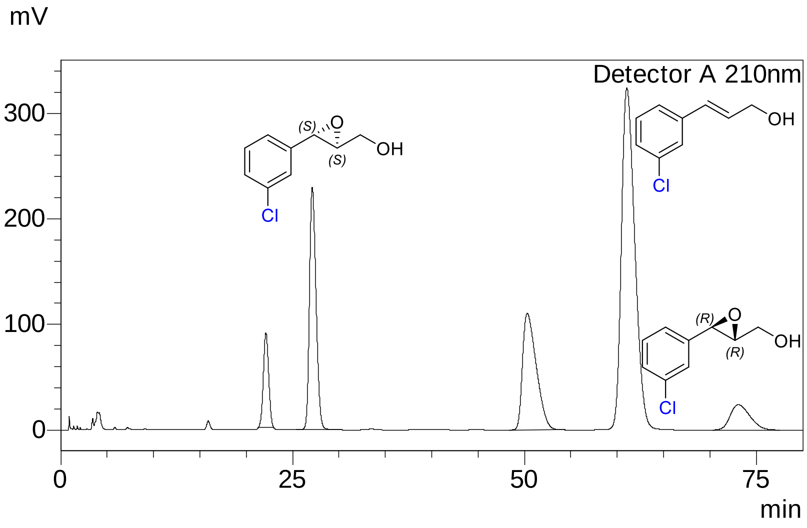

Detector A 210nm

Peak#	Ret. Time	Area	Area%
1	22,119	3562580	11,932
2	27,119	11342293	37,988
3	50,307	11419448	38,246
4	73,065	3533321	11,834
Total		29857642	100,000

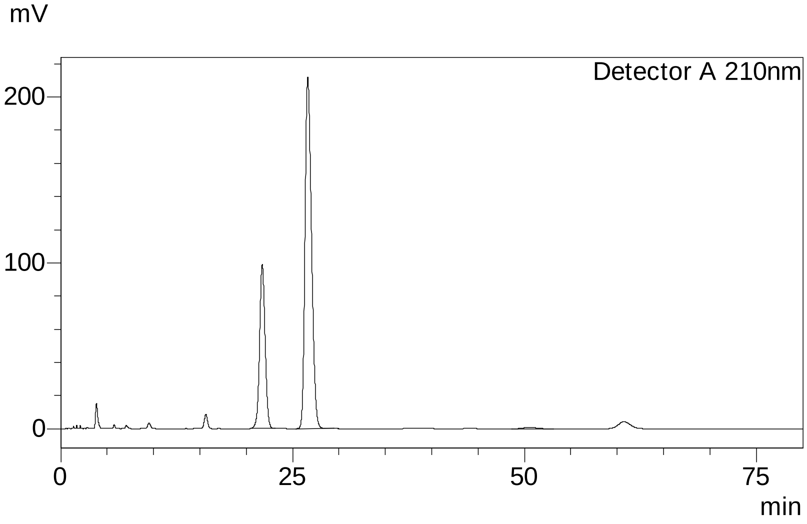

Detector A 210nm

Peak#	Ret. Time	Area	Area%
1	21,721	4073344	28,204
2	26,635	10282041	71,192
3	50,598	87230	0,604
Total		14442615	100,000

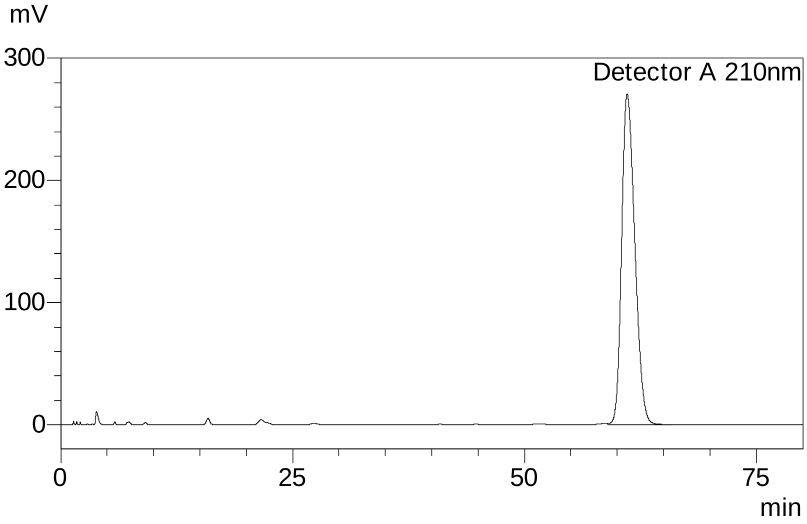

Detector A 210nm

Peak#	Ret. Time	Area	Area%
1	61,077	26762245	100,000
Total		26762245	100,000

Figure S18. Chiral reverse-phase HPLC chromatogram of product **4c**. From top to bottom are as follows: chemically synthesized racemic product and authentic (*E*)-3-(3-chlorophenyl)prop-2-en-1-ol; enzymatic product synthesized with DERA-EP; control reaction (without enzyme). The assignment of the absolute configuration of enzymatic product was based on previously reported chiral reverse-phase HPLC data.[3]

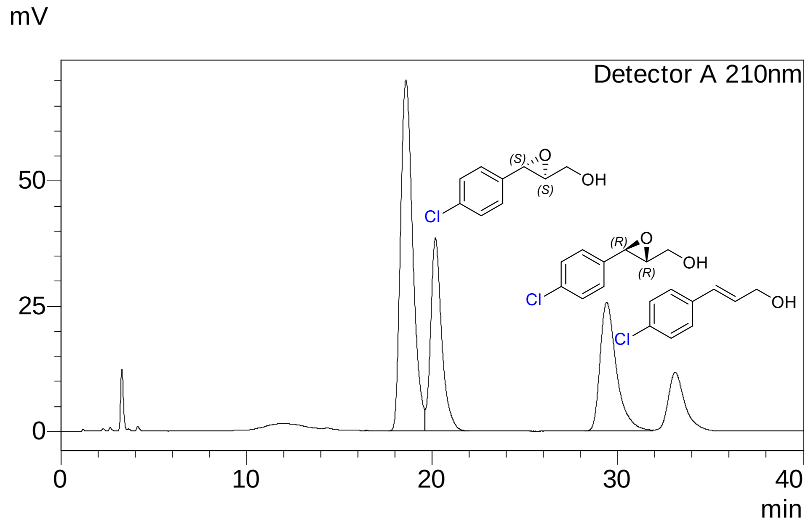

Detector A 210nm

Peak#	Ret. Time	Area	Area%
1	18,593	3207731	50,308
2	20,179	1589790	24,933
3	29,408	1578651	24,759
Total		6376173	100,000

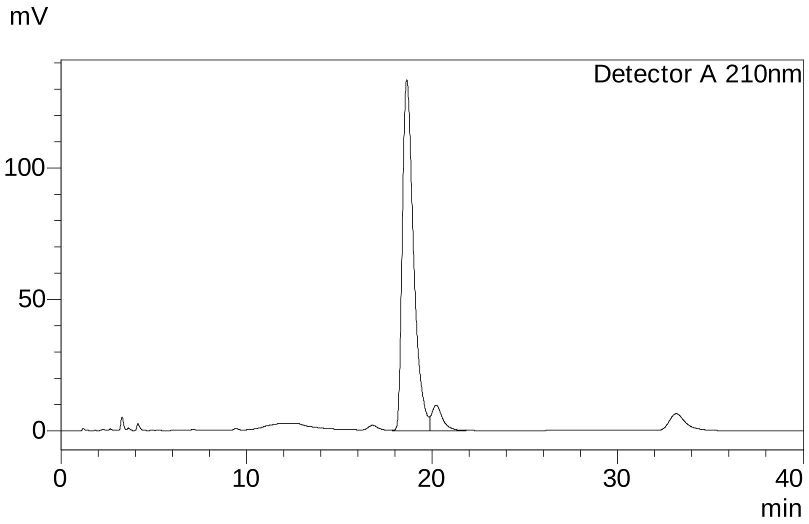

Detector A 210nm

Peak#	Ret. Time	Area	Area%
1	18,638	5662165	93,566
2	20,223	389382	6,434
Total		6051548	100,000

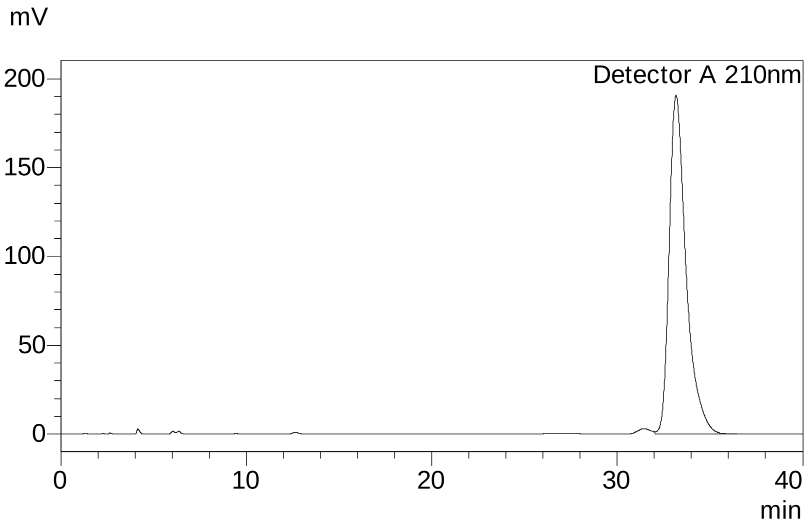

Detector A 210nm

Peak#	Ret. Time	Area	Area%
1	33,172	12068968	100,000
Total		12068968	100,000

Figure S19. Chiral reverse-phase HPLC chromatogram of product **4d**. From top to bottom are as follows: chemically synthesized racemic product and authentic (*E*)-3-(4-chlorophenyl)prop-2-en-1-ol; enzymatic product prepared with DERA-EP; control reaction (without enzyme). The assignment of the relative configuration of enzymatic product was based on previously reported chiral reverse-phase HPLC data.[3]

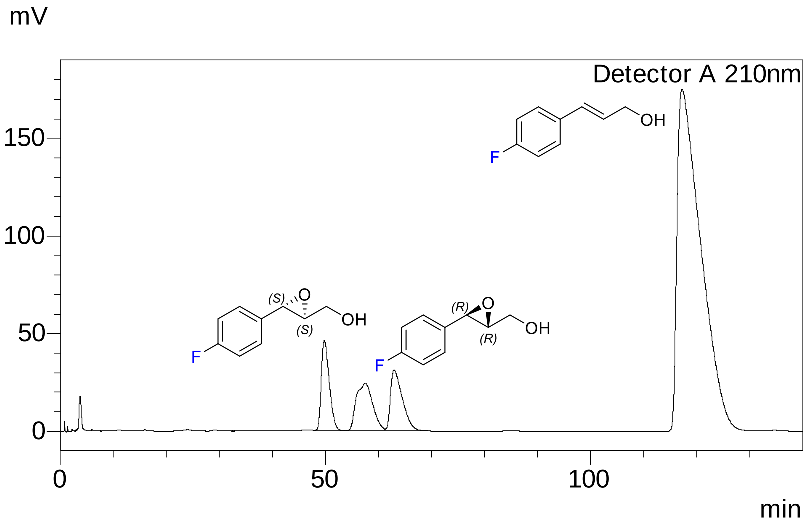

Detector A 210nm			
Peak#	Ret. Time	Area	Area%
1	49,769	4834478	32,299
2	57,518	5254343	35,104
3	62,956	4879298	32,598
Total		14968120	100,000

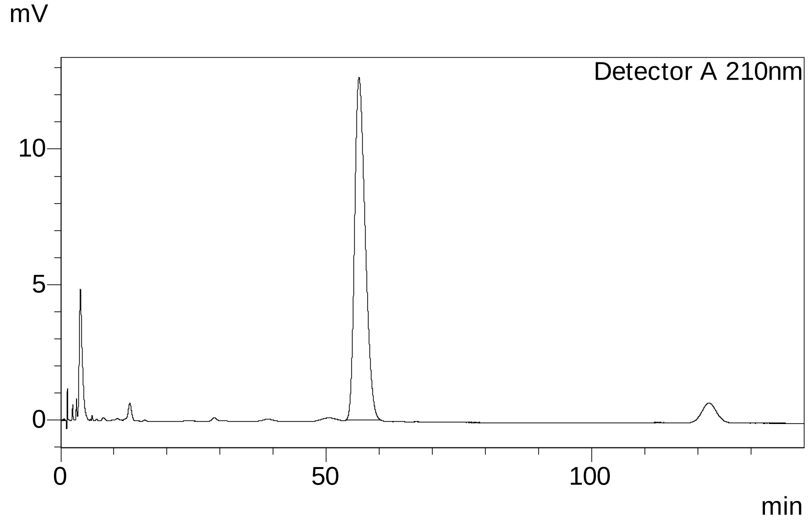

Detector A 210nm			
Peak#	Ret. Time	Area	Area%
1	56,177	1750646	100,000
Total		1750646	100,000

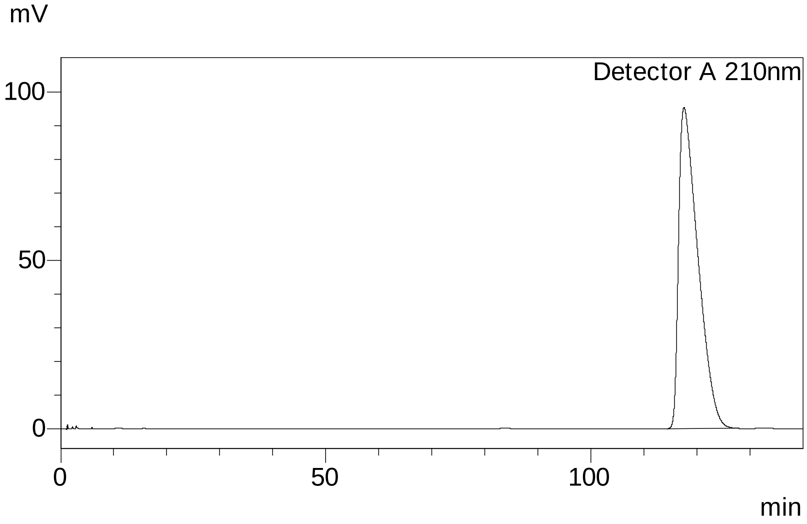

Detector A 210nm			
Peak#	Ret. Time	Area	Area%
1	117,606	24066654	100,000
Total		24066654	100,000

Figure S20. Chiral reverse-phase HPLC chromatogram of product **4e**. From top to bottom are as follows: Chemically synthesized racemic product and authentic (*E*)-3-(4-fluorophenyl)prop-2-en-1-ol; enzymatic product prepared with DERA-EP; control reaction (without enzyme). The assignment of the relative configuration of enzymatic product was based on previously reported chiral reverse-phase HPLC data.[3]

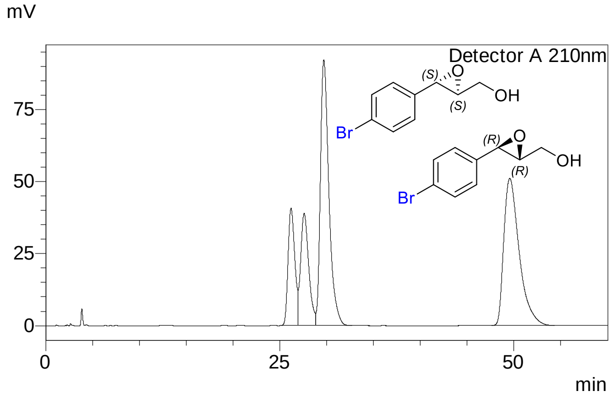

Peak#	Ret. Time	Area	Area%
1	26,203	2160587	13,454
2	27,604	2248039	13,999
3	29,704	5836368	36,343
4	49,550	5814004	36,204
Total		16058997	100,000

Detector A 210nm

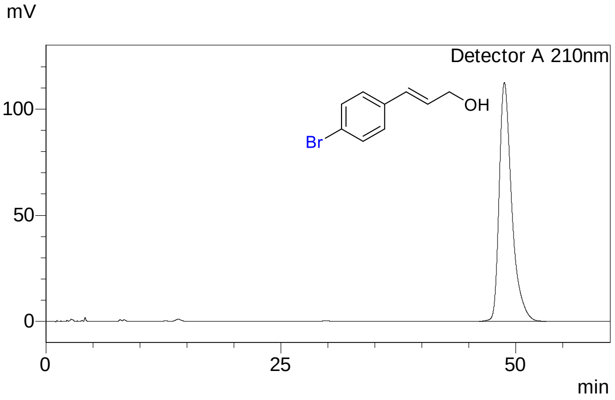

Detector A 210nm

Peak#	Ret. Time	Area	Area%
1	48,767	10427637	100,000
Total		10427637	100,000

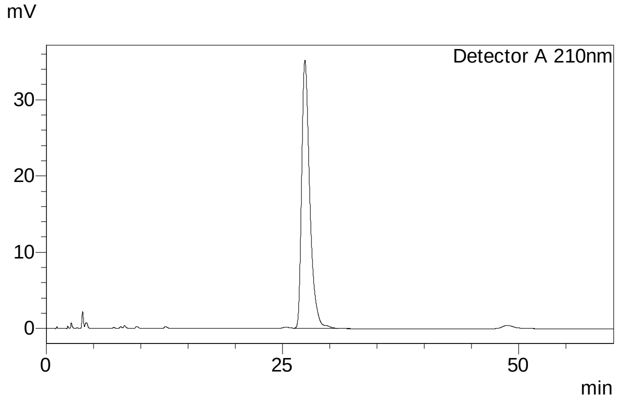

Detector A 210nm

Peak#	Ret. Time	Area	Area%
1	27,380	2100239	100,000
Total		2100239	100,000

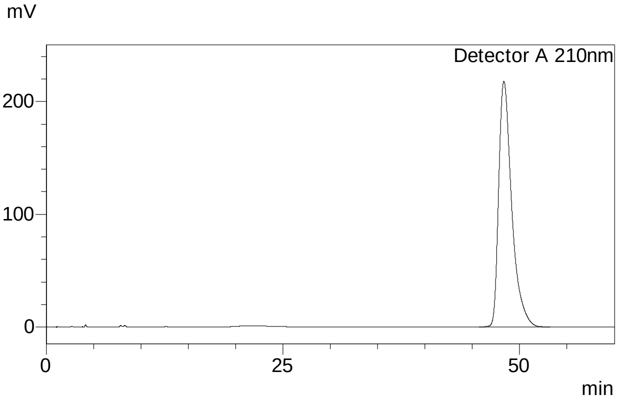

Detector A 210nm

Peak#	Ret. Time	Area	Area%
1	48,331	20870017	100,000
Total		20870017	100,000

Figure S21. Chiral reverse-phase HPLC chromatogram of product **4f**. From top to bottom are as follows: chemically synthesized racemic product; authentic reference compound (*E*)-3-(4-bromophenyl)prop-2-en-1-ol; enzymatic product prepared with DERA-EP; control reaction (without enzyme). The assignment of the relative configuration of enzymatic product was based on previously reported chiral reverse-phase HPLC data.[3]

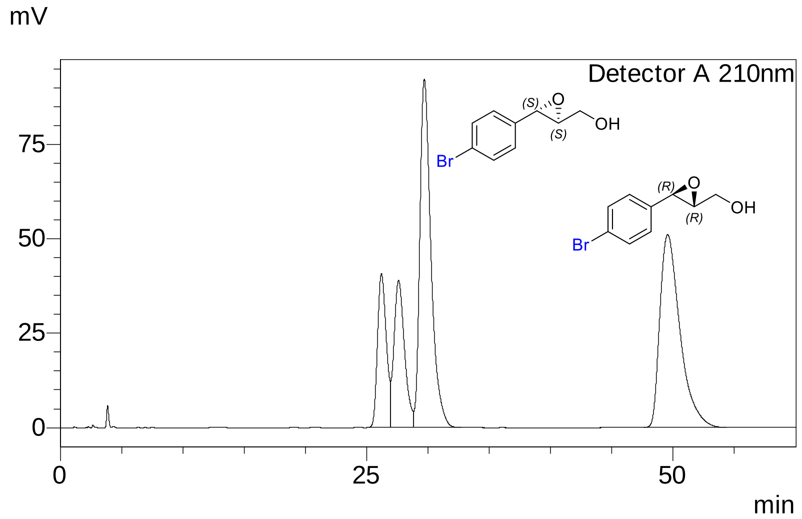

Detector A 210nm

Peak#	Ret. Time	Area	Area%
1	26,203	2160587	13,454
2	27,604	2248039	13,999
3	29,704	5836368	36,343
4	49,550	5814004	36,204
Total		16058997	100,000

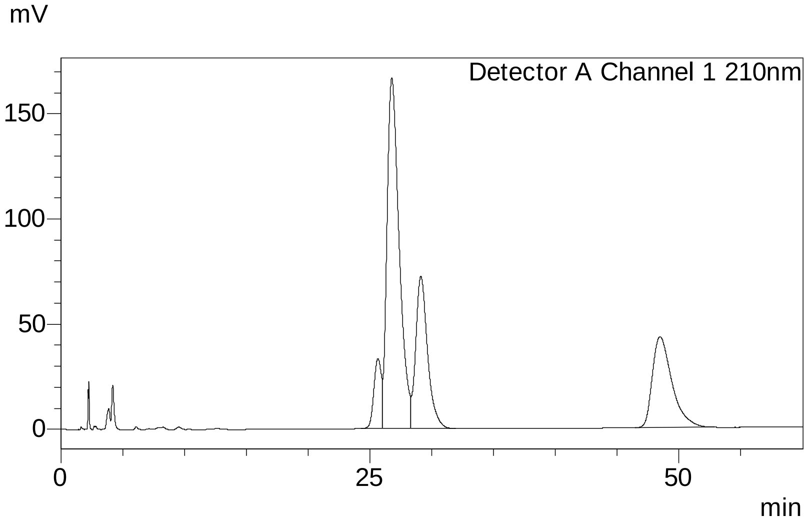

Detector A Channel 1 210nm

Peak#	Ret. Time	Area	Area%
1	25,651	1384872	6,167
2	26,769	11460240	51,032
3	29,118	4835700	21,533
4	48,463	4776011	21,268
Total		22456823	100,000

Figure S22. Chiral reverse-phase HPLC chromatogram of product **4f**. From top to bottom are as follows: chemically synthesized racemic product; racemic product spiked with enzymatic product prepared with DERA-EP. The assignment of the relative configuration of enzymatic product was based on previously reported chiral reverse-phase HPLC data.[3]

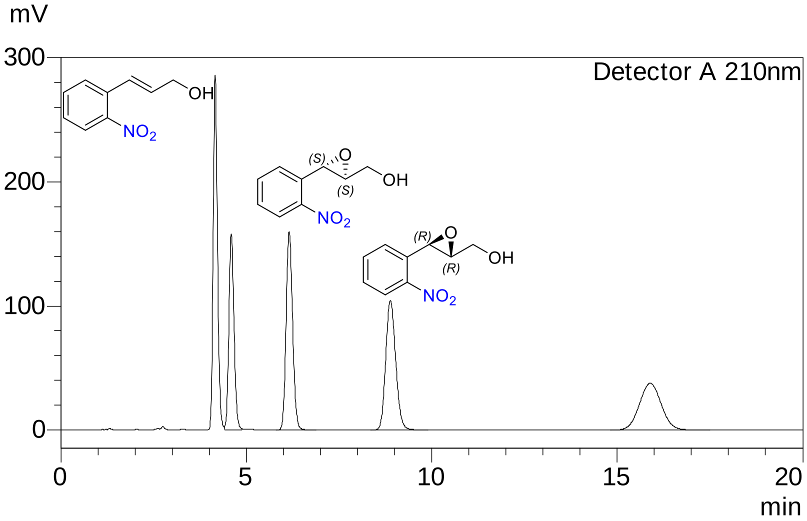

Detector A 210nm

Peak#	Ret. Time	Area	Area%
1	4,597	1535196	22,037
2	6,158	1951729	28,017
3	8,885	1943614	27,900
4	15,890	1535780	22,046
Total		6966319	100,000

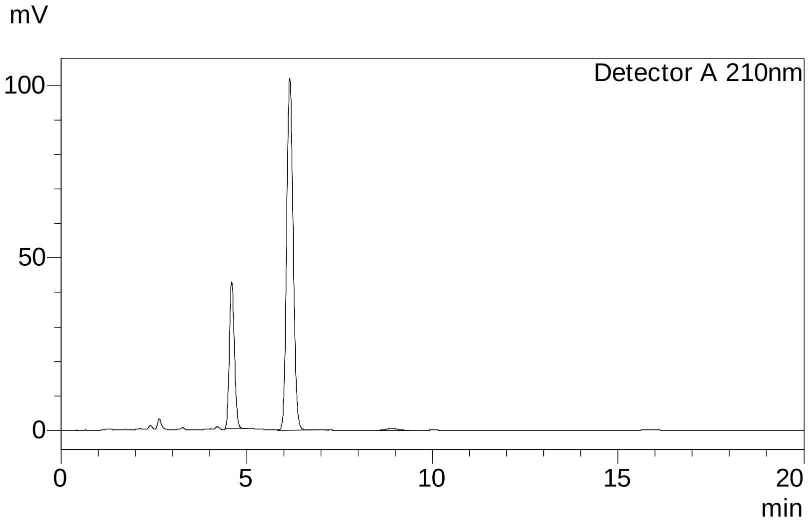

Detector A 210nm

Peak#	Ret. Time	Area	Area%
1	4,600	404461	24,414
2	6,162	1241712	74,952
3	8,904	10507	0,634
Total		1656680	100,000

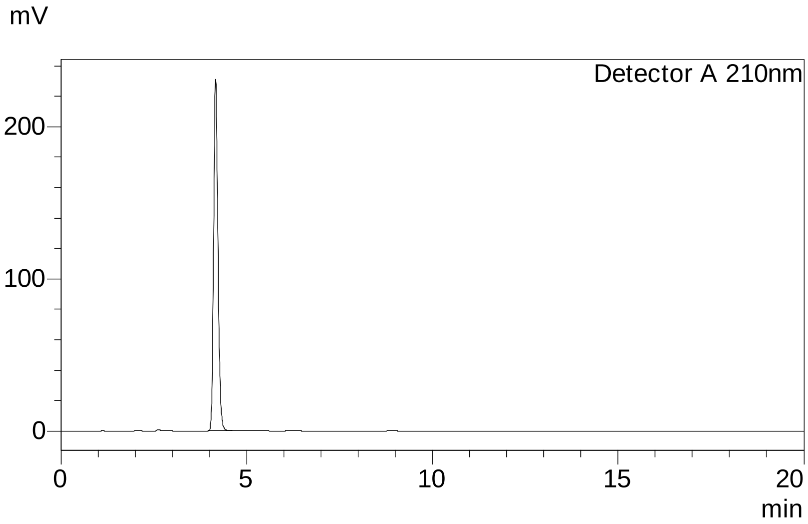

Detector A 210nm

Peak#	Ret. Time	Area	Area%
1	4,166	1883008	100,000
Total		1883008	100,000

Figure S23. Chiral reverse-phase HPLC chromatogram of product **4g**. From top to bottom are as follows: chemically synthesized racemic product and authentic (*E*)-3-(2-nitrophenyl)prop-2-en-1-ol; enzymatic product prepared with DERA-EP, control reaction (without enzyme). The assignment of the absolute configuration of enzymatic product was based on previously reported chiral reverse-phase HPLC data.[3]

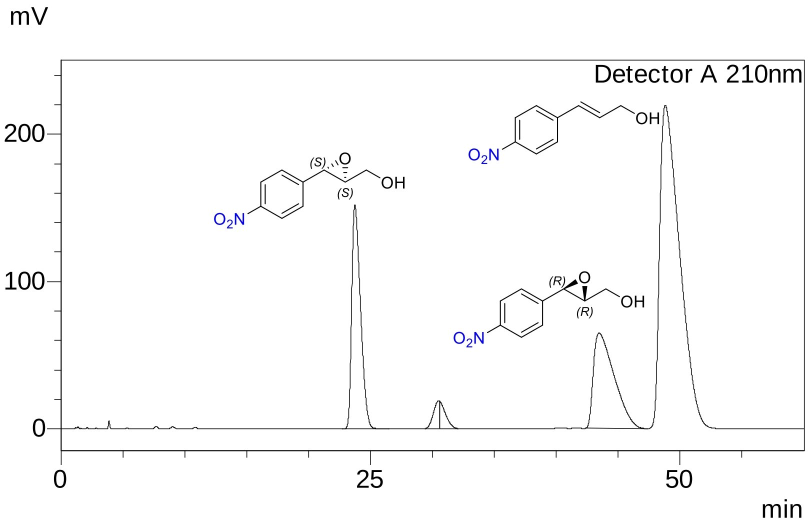

Detector A 210nm

Peak#	Ret. Time	Area	Area%
1	23,743	7536706	46,157
2	30,507	624135	3,822
3	30,579	616672	3,777
4	43,465	7550836	46,244
Total		16328349	100,000

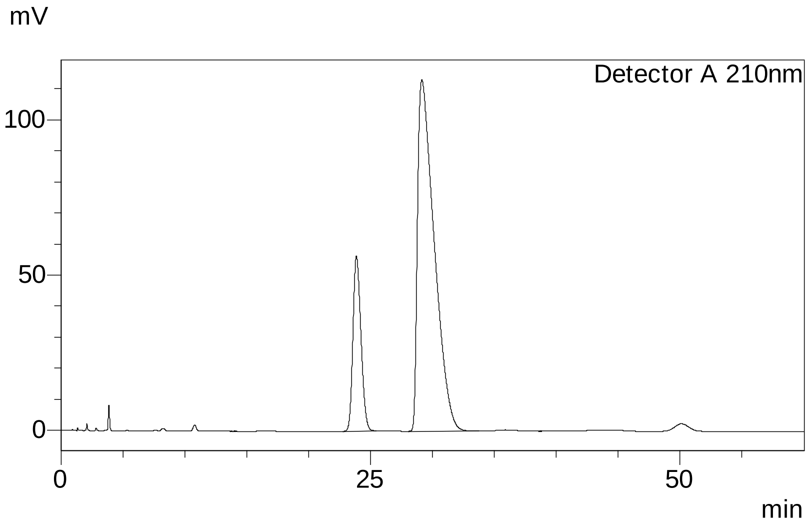

Detector A 210nm

Peak#	Ret. Time	Area	Area%
1	23,863	2542256	19,363
2	29,145	10587240	80,637
Total		13129496	100,000

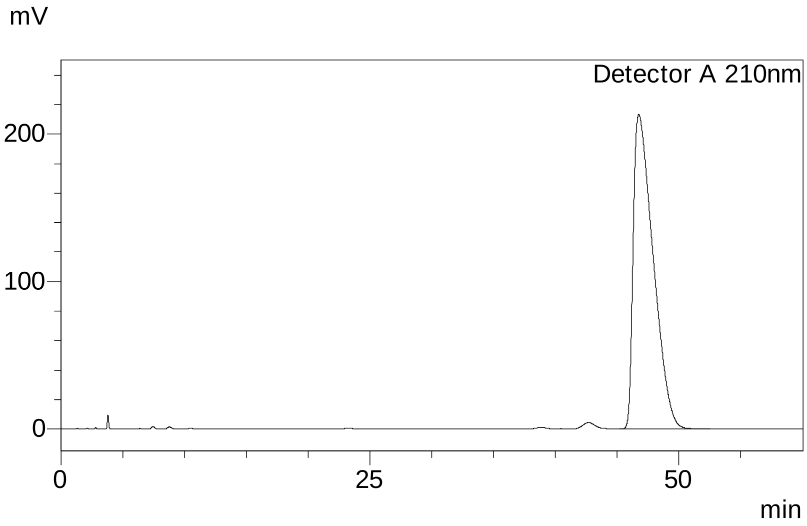

Detector A 210nm

Peak#	Ret. Time	Area	Area%
1	46,746	23904959	100,000
Total		23904959	100,000

Figure S24. Chiral reverse-phase HPLC chromatogram of product **4h**. From top to bottom are as follows: chemically synthesized racemic product and authentic (*E*)-3-(4-nitrophenyl)prop-2-en-1-ol; enzymatic product prepared with DERA-EP, control reaction (without enzyme). The assignment of the relative configuration of enzymatic product was based on previously reported chiral reverse-phase HPLC data.[3]

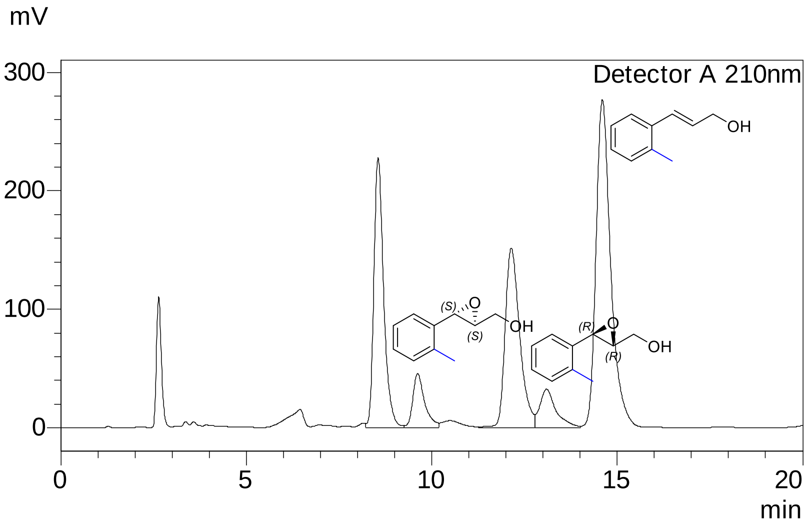

Detector A 210nm

Peak#	Ret. Time	Area	Area%
1	8,554	4168244	40,319
2	9,620	1016914	9,836
3	12,144	4162792	40,266
4	13,093	990229	9,578
Total		10338178	100,000

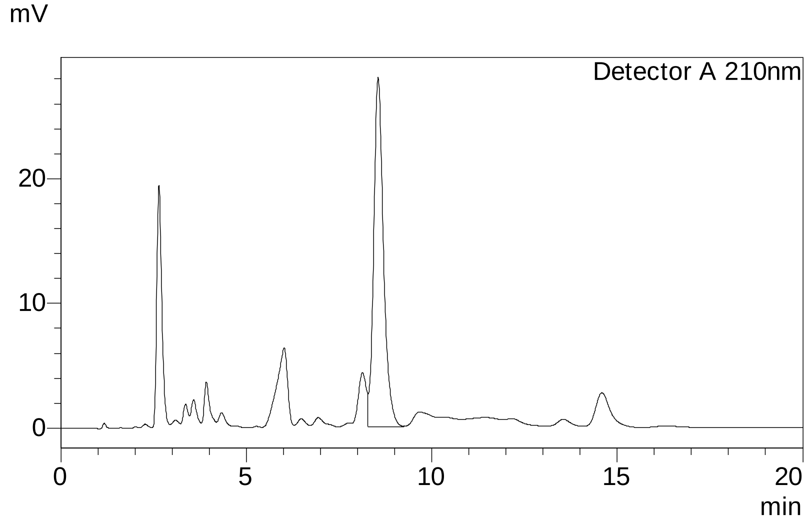

Detector A 210nm

Peak#	Ret. Time	Area	Area%
1	8,554	510393	100,000
Total		510393	100,000

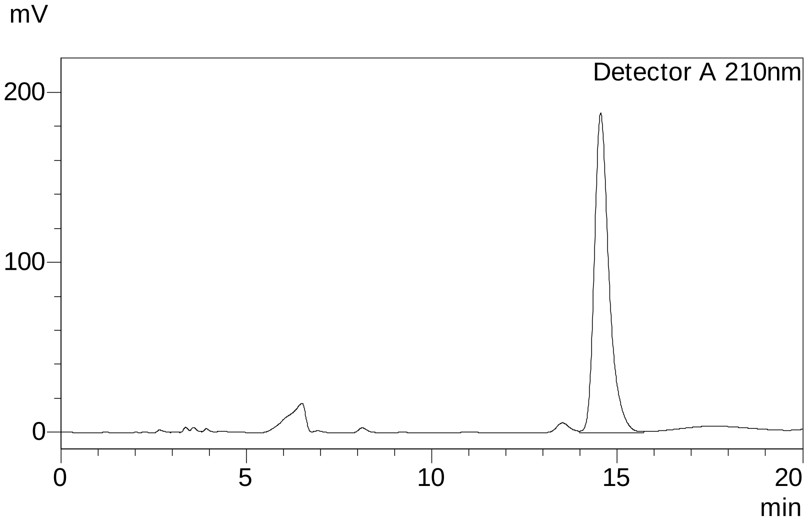

Detector A 210nm

Peak#	Ret. Time	Area	Area%
1	14,554	5166963	100,000
Total		5166963	100,000

Figure S25. Chiral reverse-phase HPLC chromatogram of product **4i**. From top to bottom are as follows: chemically synthesized racemic product and authentic (*E*)-3-(*o*-tolyl)prop-2-en-1-ol; enzymatic product prepared with DERA-EP; control reaction (without enzyme). The assignment of the relative configuration of enzymatic product was based on previously reported chiral reverse-phase HPLC data.[3]

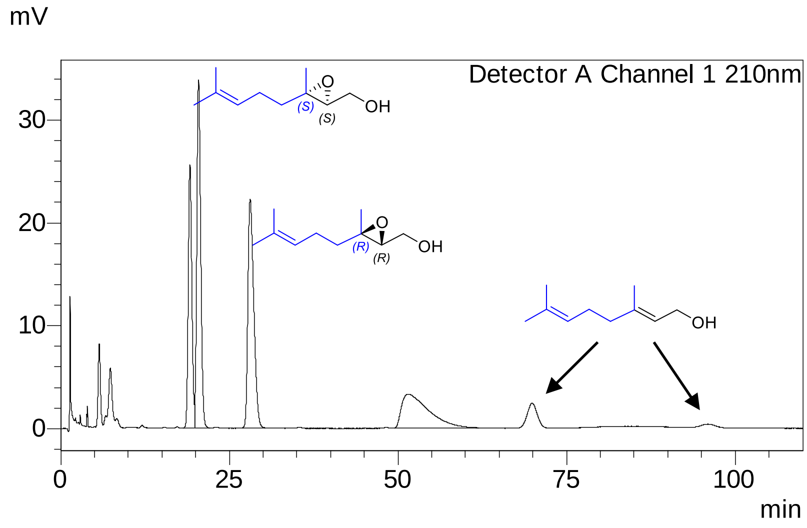

Peak#	Ret. Time	Area	Area%
1	19,150	941131	20,578
2	20,424	1367634	29,903
3	28,077	1349205	29,501
4	51,593	915523	20,018
Total		4573493	100,000

Detector A Channel 1 210nm

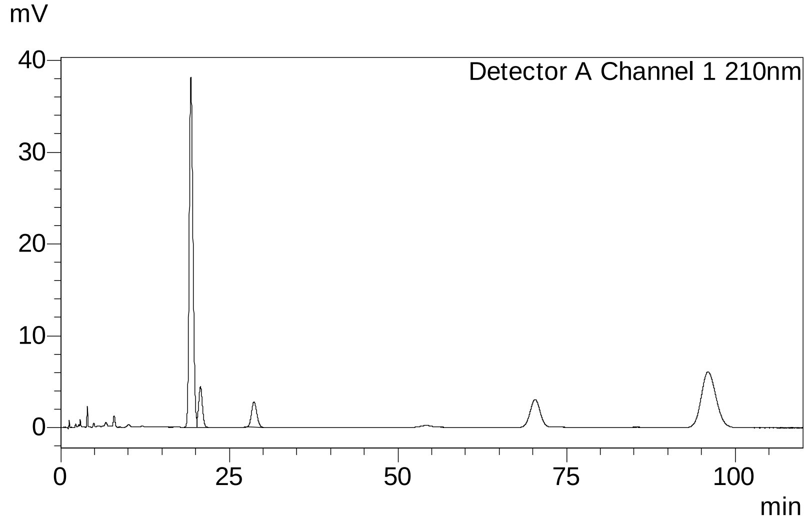

Peak#	Ret. Time	Area	Area%
1	19,293	1444548	81,697
2	20,717	171696	9,710
3	28,649	151934	8,593
Total		1768178	100,000

Detector A Channel 1 210nm

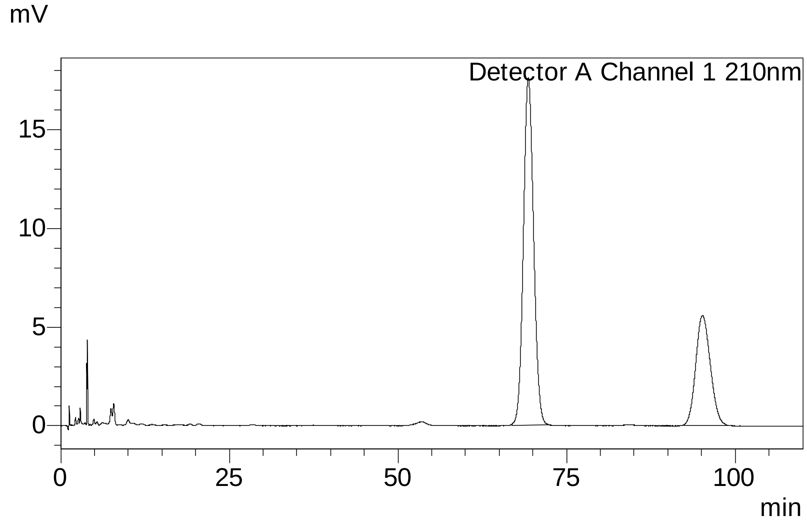

Peak#	Ret. Time	Area	Area%
1	69,322	1846686	68,816
2	95,165	836818	31,184
Total		2683504	100,000

Detector A Channel 1 210nm

Figure S26. Chiral reverse-phase HPLC chromatogram of product **4j**. From top to bottom are as follows: chemically synthesized racemic product and authentic (*E/Z*)-3,7-dimethylocta-2,6-dien-1-ol; enzymatic product prepared with DERA-EP; control reaction (without enzyme). The assignment of the relative configuration of enzymatic product was based on previously reported chiral reverse-phase HPLC data.[3]

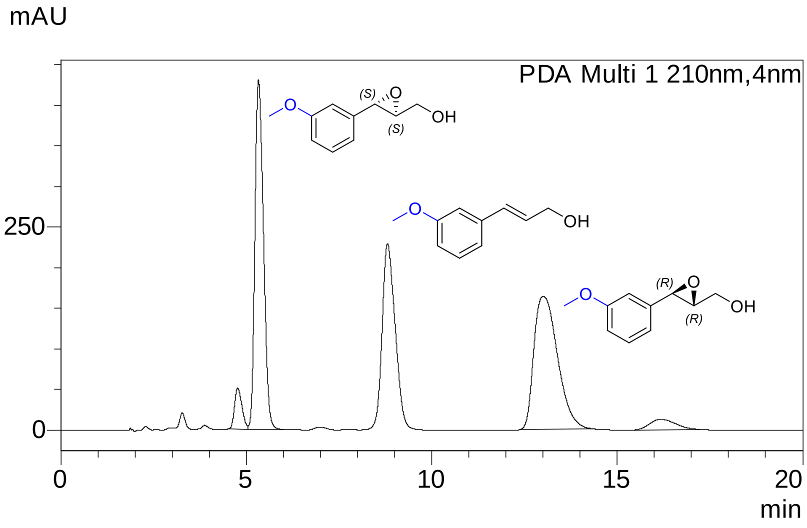

PDA Ch1 210nm

Peak#	Ret. Time	Area	Area%
1	4,769	706314	4,559
2	5,330	6891114	44,478
3	13,008	7278285	46,977
4	16,189	617495	3,986
Total		15493208	100,000

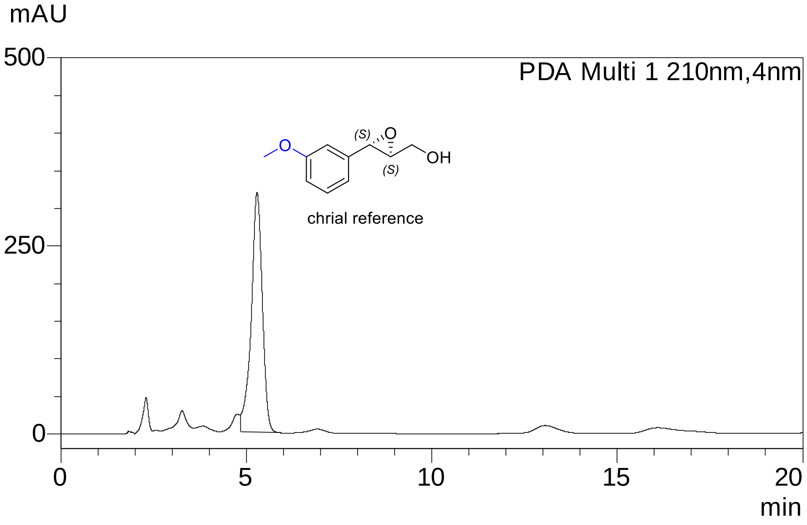

PDA Ch1 210nm

Peak#	Ret. Time	Area	Area%
1	5,292	6636936	100,000
Total		6636936	100,000

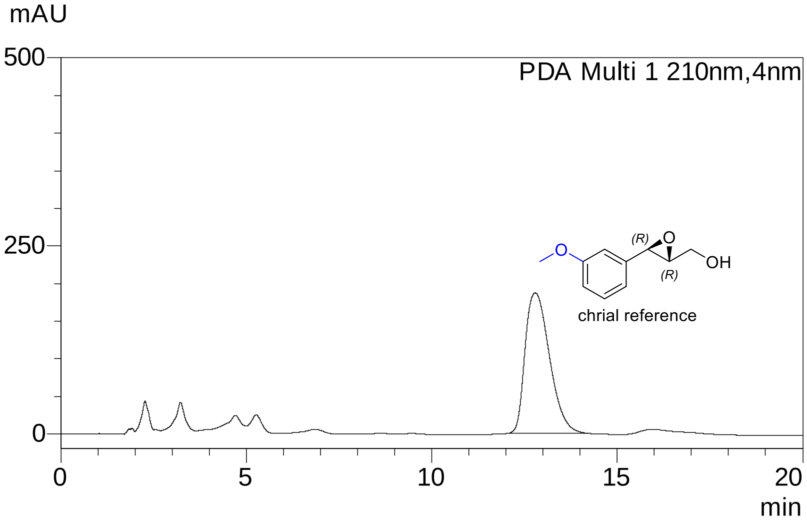

PDA Ch1 210nm

Peak#	Ret. Time	Area	Area%
1	12,790	8523675	100,000
Total		8523675	100,000

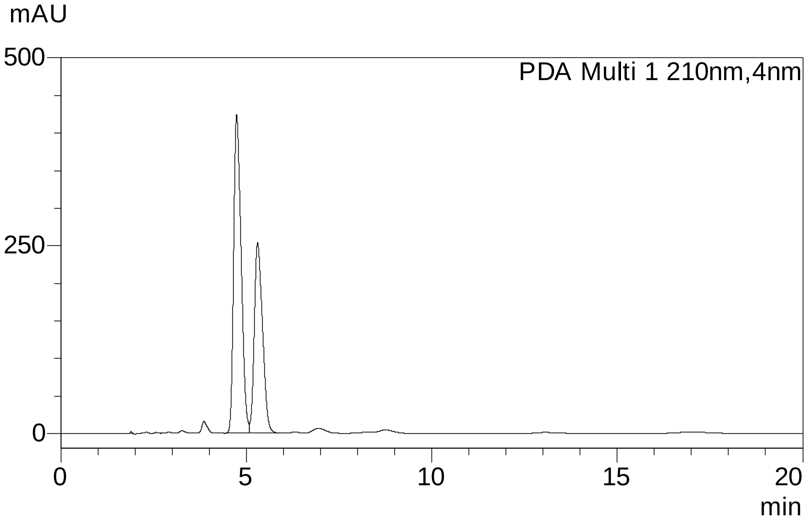

PDA Ch1 210nm

Peak#	Ret. Time	Area	Area%
1	4,741	5950529	60,368
2	5,307	3906489	39,632
Total		9857019	100,000

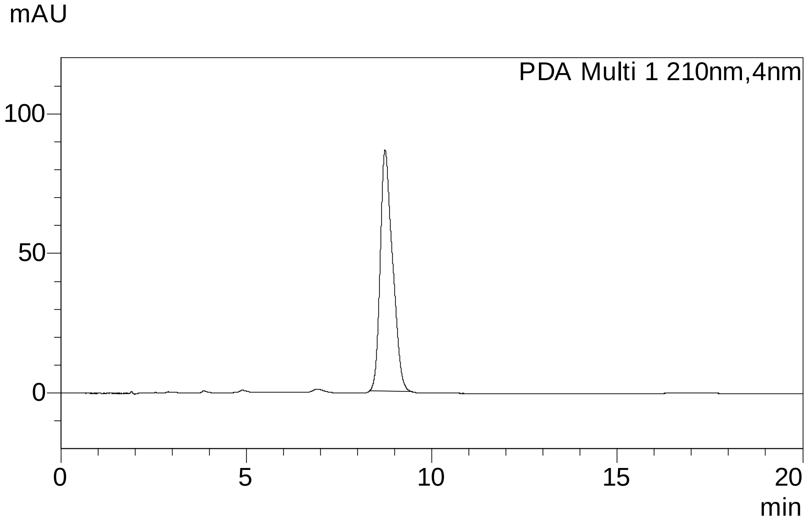

PDA Ch1 210nm

Peak#	Ret. Time	Area	Area%
1	8,743	1997491	100,000
Total		1997491	100,000

Figure S27. Chiral reverse-phase HPLC chromatogram of product **4k**. From top to bottom are as follows: chemically synthesized racemic product and authentic (*E*)-3-(3-methoxyphenyl)prop-2-en-1-ol; authentic chiral reference compound (2*S*, 3*S*)-**4k**; authentic chiral reference compound (2*R*, 3*R*)-**4k**; enzymatic product synthesized with DERA-EP; and control reaction (without enzyme).

16. Supplementary References

[1] H. Chen, H. Jiang, C. Cai, J. Dong, W. Fu, *Org. Lett.* **2011**, *13*, 992–994.

[2] A. Kunzendorf, G. Xu, M. Saifuddin, T. Saravanan, G. J. Poelarends, *Angew. Chemie Int. Ed.* **2021**, *60*, 24059–24063.

[3] G. Xu, M. Crotti, T. Saravanan, K. M. Kataja, G. J. Poelarends, *Angew. Chemie Int. Ed.* **2020**, *59*, 10374–10378.

[4] A. Kunzendorf, G. Xu, J. J. H. van der Velde, H. J. Rozeboom, A. W. H. Thunnissen, G. J. Poelarends, *ACS Catal.* **2021**, *11*, 13236–13243.

[5] S. Voutilainen, M. Heinonen, M. Andberg, E. Jokinen, H. Maaheimo, J. Pääkkönen, N. Hakulinen, J. Rouvinen, H. Lähdesmäki, S. Kaski, J. Rousu, M. Penttilä, A. Koivula, *Appl. Microbiol. Biotechnol.* **2020**, *104*, 10515–10529.

[6] R. M. P. Siloto, R. J. Weselake, *Biocatal. Agric. Biotechnol.* **2012**, *1*, 181–189.

[7] T. A. Kunkel, *Proc. Natl. Acad. Sci.* **1985**, *82*, 488–492.

[8] L. Biewenga, M. Crotti, M. Saifuddin, G. J. Poelarends, *ACS Omega* **2020**, *5*, 2397–2405.

[9] M. Sigmund, G. Xu, E. Grandi, G. J. Poelarends, *Chem. – A Eur. J.* **2022**, *28*, e202201651.

[10] B. O. Burek, S. Bormann, F. Hollmann, J. Z. Bloh, D. Holtmann, *Green Chem.* **2019**, *21*, 3232–3249.

[11] M. Marigo, J. Franzén, T. B. Poulsen, W. Zhuang, K. A. Jørgensen, *J. Am. Chem. Soc.* **2005**, *127*, 6964–6965.

[12] W. Kabsch, *Acta Crystallogr. Sect. D Biol. Crystallogr.* **2010**, *66*, 125–132.

[13] P. R. Evans, G. N. Murshudov, *Acta Crystallogr. Sect. D Biol. Crystallogr.* **2013**, *69*, 1204–1214.

[14] M. D. Winn, C. C. Ballard, K. D. Cowtan, E. J. Dodson, P. Emsley, P. R. Evans, R. M. Keegan, E. B. Krissinel, A. G. W. Leslie, A. McCoy, S. J. McNicholas, G. N. Murshudov, N. S. Pannu, E. A. Potterton, H. R. Powell, R. J. Read, A. Vagin, K. S. Wilson, *Acta Crystallogr. Sect. D Biol. Crystallogr.* **2011**, *67*, 235–242.

[15] A. J. McCoy, R. W. Grosse-Kunstleve, P. D. Adams, M. D. Winn, L. C. Storoni, R. J. Read, *J. Appl. Crystallogr.* **2007**, *40*, 658–674.

[16] P. Emsley, B. Lohkamp, W. G. Scott, K. Cowtan, *Acta Crystallogr. Sect. D Biol. Crystallogr.* **2010**, *66*, 486–501.

[17] G. N. Murshudov, P. Skubák, A. A. Lebedev, N. S. Pannu, R. A. Steiner, R. A. Nicholls, M. D. Winn, F. Long, A. A. Vagin, *Acta Crystallogr. Sect. D Biol. Crystallogr.* **2011**, *67*, 355–367.

[18] F. Long, R. A. Nicholls, P. Emsley, S. Gražulis, A. Merkys, A. Vaitkus, G. N. Murshudov, *Acta Crystallogr. Sect. D Struct. Biol.* **2017**, *73*, 112–122.

[19] C. J. Williams, J. J. Headd, N. W. Moriarty, M. G. Prisant, L. L. Videau, L. N. Deis, V. Verma, D. A. Keedy, B. J. Hintze, V. B. Chen, S. Jain, S. M. Lewis, W. B. Arendall, J.

Snoeyink, P. D. Adams, S. C. Lovell, J. S. Richardson, D. C. Richardson, *Protein Sci.* **2018**, *27*, 293–315.

[20] C. Sparr, W. B. Schweizer, H. M. Senn, R. Gilmour, *Angew. Chemie Int. Ed.* **2009**, *48*, 3065–3068.

[21] T. Nemoto, T. Ohshima, M. Shibasaki, *J. Am. Chem. Soc.* **2001**, *123*, 9474–9475.

[22] B. Wang, X. Wu, O. A. Wong, B. Nettles, M. Zhao, D. Chen, Y. Shi, *J. Org. Chem.* **2009**, *74*, 3986–3989.
